# Vitamin D receptor prevents tumour development by regulating the Wnt/β-catenin signalling pathway in human colorectal cancer

**DOI:** 10.1186/s12885-023-10690-z

**Published:** 2023-04-12

**Authors:** Jie Yu, Qi Sun, Yi Hui, Jinping Xu, Pancheng Shi, Yu Chen, Yunzhao Chen

**Affiliations:** 1 Department of Pathology, The People’s Hospital of Suzhou New District, No. 95, Huashan Road, High Tech Zone, Suzhou, Jiangsu Prov China; 2 Department of Pathology, The Affiliated Drum Tower Hospital of Nanjing University Medical School, Nanjing, China; 3 Department of Pathology, Zhejiang Provincial People’s Hospital, Hangzhou, China

**Keywords:** Colorectal cancer, Invasion, Apoptosis, Vitamin D receptor, Wnt/β-catenin, Cyclin D1

## Abstract

**Background:**

Colorectal cancer (CRC) is a common disease threatening human lives worldwide, and vitamin D receptor (VDR) contributes protective roles in this disease. However, the molecular mechanisms underlying VDR protection in CRC progression require further investigation.

**Methods:**

In this study, we statistically analyzed the relationship between VDR expression and CRC development in patients and detected invasion and apoptosis in CRC cells with VDR overexpression and interference. We also detected the expression of key genes involved in Wnt/β-catenin signalling (β-catenin, lymphoid enhancer factor (LEF)-1 and cyclin D1) in SW480 cells and nude mice injected with VDR-overexpressing SW480 cells and observed tumour development. Additionally, we performed Co-immunoprecipitation (Co-IP) and glutathione-S-transferase (GST) pull-down assays to identify the protein interactions of VDR with β-catenin, dual luciferase (LUC) and chromatin immunoprecipitation (ChIP) to detect the activation of LEF-1 by VDR.

**Results:**

The VDR level was closely related to the development and prognosis of CRC patients. VDR overexpression inhibited invasion but promoted apoptosis in cancer cells. β-catenin shRNA contributed oppositely to cancer cell activity with VDR shRNA. Additionally, VDR interacted with β-catenin at the protein level and blocked its nuclear accumulation. VDR regulated the expression of β-catenin, cyclin D1 and LEF-1 and directly activated LEF-1 transcription in vitro. Furthermore, nude mice injected with VDR-overexpressing SW480 cells revealed suppression of tumour growth and decreased expression of β-catenin, cyclin D1 and LEF-1.

**Conclusions:**

This study indicated that VDR protected against CRC disease in humans by inhibiting Wnt/β-catenin signalling to control cancer cell invasion and apoptosis, providing new evidence to explore VDR biomarkers or agonists for CRC patient diagnosis and treatment.

**Supplementary Information:**

The online version contains supplementary material available at 10.1186/s12885-023-10690-z.

## Background

CRC is a commonly diagnosed cancer and a global health issue [1, 2]. The incidence and mortality of CRC rank third among common cancers worldwide [3, 4]. According to data from the World Health Organization (WHO), 140 million new CRC patients were diagnosed worldwide, and approximately 700,000 of them died of this disease in 2012 [4]. Thirty percent of patients undergoing surgery, radiotherapy and chemotherapy relapse within 3 years [5]. Despite progress in early screening and systemic therapy, most CRC patients die from tumour metastasis [6]. Thus, studying the molecular mechanism of CRC metastasis is valuable to improve the prognosis of patients.

VDR belongs to the steroid hormone receptor superfamily and is a nuclear transcription factor [7, 8]. It forms a complex with the VD metabolite 1α,25(OH)_2_vitamin D_3_ (1α,25(OH)_2_D_3_), which plays important roles in the expression of numerous genes, maintaining calcium/phosphate homeostasis, regulating cellular proliferation and differentiation, and organizing the immune response [7, 9–11]. VDR is universally present in most nucleated cells at variable concentrations and is expressed in virtually all tissues, including bone, colon, breast, ovary, lung, kidney, immune cells and even cancer cells [12–14], exerting a broad spectrum of functions. Recent studies have found that VDR is abnormally expressed in various malignant tumours, such as prostate cancer, ovarian cancer, and breast cancer [14, 15]. Although VDR expression has already been reported to be associated with prognosis of CRC [16, 17], additional knowledge on its functional significance and the prognostic impact in CRC were needed.

Wnt signalling controls a series of biological developments in animals [18, 19]. Aberrant Wnt signalling is connected to extensive pathologies and has been described in many cancer cases [18, 20]. Wnt signalling cascades are divided into two types: β-catenin-dependent and β-catenin-independent. In recent years, a model of the Wnt/β-catenin signalling pathway has been well defined. The absence of Wnt proteins results in β-catenin phosphorylation and degradation in the cytoplasm by the destruction complex containing adenomatous polyposis coli (APC), Axin, glycogen synthase kinase-3beta (GSK3beta) and casein kinase (CK)1α. These events prevent β-catenin deposition in the nucleus and repress gene activation by the repressive complex containing T-cell factor (TCF)/LEF. By contrast, the Wnt/β-catenin signalling pathway is activated when Wnt-secreted proteins are bound to the Frizzled (Fzd) receptor, leading to inactivation of the destruction complex by the phosphorylation of GSK3beta and CK1α and nuclear accumulation of β-catenin. Subsequently, β-catenin forms an active complex with TCF/LEF proteins and leads to a transcriptional switch related to multiple biological processes [20–22]. Abnormal expression of the Wnt/β-catenin signalling pathway is related to the occurrence and development of various tumours, such as colorectal cancer, gastric cancer, oesophageal cancer, and nasopharyngeal cancer [23–26]. Almost all the CRC patients harbored mutations in the Wnt/β-catenin signalling pathway, indicating the importance of this pathway in CRC [27]. Increased activity of the Wnt signalling pathway causes the accumulation of excessive β-catenin in cells, intestinal epithelial cells lose homeostasis and the epithelial structure disappears, promoting tumour infiltration and metastasis. Additionally, recent studies have shown that low VDR protein expression promotes the nuclear deposition of β-catenin in the tumour cells of APC-mutated mice [28]. Although novel insights into the interaction of VDR and the Wnt/β-catenin pathway exerting antitumour effects have been obtained [29, 30], the molecular mechanisms underlying VDR regulating invasion and apoptosis through the Wnt/β-catenin signalling pathway in CRC remain unclear and must be further elucidated.

In this study, we statistically analyzed 188 human CRC tissue samples of cases and 134 human colorectal normal epithelial tissue samples. We designed lentivirus-delivered VDR silencing and overexpression constructs in SW480 cells, detected the expression of key genes in Wnt/β-catenin signalling, and observed tumour growth in nude mice injected with VDR-OE SW480 cells. VDR was closely related to CRC progression and contributed to antitumour effects by inhibiting Wnt/β-catenin signalling. Additionally, VDR interacted with β-catenin at the protein level and directly activated LEF-1 transcription in vitro. Taken together, VDR protects against CRC by regulating the Wnt/β-catenin signalling pathway. Strategies to maintain VDR expression in CRC patients are promising to prevent and treat cancers in the future.

## Methods

### Patients

The CRC tissues used in this experiment were collected from tissue specimens at the Department of Pathology, People’s Hospital of Suzhou High-tech Zone from 2009 to 2021. The CRC tissues were diagnosed by senior pathologists; 188 samples were colorectal cancer tissues, and 134 samples were colorectal normal epithelial tissues. The diagnostic criteria referred to the 2010 edition of ‘WHO Classification Tumours of the Digestive System’ and the 2009 7th edition of ‘the American Joint Committee on Cancer, AJCC’ tumour lymph node metastasis (TNM) staging standards. This study was conducted with written informed consent obtained from the individual patients and was approved by the ethics committees of People’s Hospital of Suzhou High-tech Zone.

### Cell culture

The human CRC cell line SW480 was purchased from the Cell Bank of the Chinese Academy of Sciences. SW480 cells were resuspended in Dulbecco’s modified Eagle’s medium (DMEM) containing 10% foetal bovine serum and 1% penicillin-streptomycin solutions and were incubated with 5% CO_2_ at 37 °C. We replaced the culture medium with fresh medium every 2–3 days until the cells grew to approximately 90% of the culture flask area and conducted cell experiments. Well-growing cells at passages 3–10 were chosen for subsequent experiments.

### Cell transfection

A lentiviral vector encoding shRNAs for VDR and β-catenin were developed by Biostorms (Suzhou, China). The VDR sequence (5′-GGTCAGTTACAGCATCCAA-3′) and β-catenin sequence (5′-GCACAAGAATGGATCACAA-3′) were incorporated into the pLVH1-GFP vector, respectively. To produce lentivirus containing VDR or β-catenin, HEK-293 T cells were hybridized with the pLV-VDR or pLV-β-catenin plasmid using the Virapower Packing Mix and Lipofectamine 2000 (Thermo, USA). A vector for the overexpression of VDR was obtained from Biostorms. Chemical modifications of pre-VDR were included for use in selection and to improve the stability of the guide chain. A negative control was prepared using a nonsense oligonucleotide. After the prepared lentivirus were infected into SW480 cells, they were observed under a fluorescence microscope (CX33; Olympus).

### Animal experiment

The animal experiment in this study was approved by the institutional ethics committee of Soochow University. BALB/C nude mice were purchased from Vital River (Beijing). OE-VDR SW480 cells and empty controls were injected into the mice. Thirteen days after the injection, the tumours were separated and measured using an electronic balance and Vernier calipers.

### Quantitative real-time PCR (qRT–PCR)

Total RNA was extracted using TRIzol reagent (Invitrogen, US) following the manufacturer’s instructions. Reverse transcription was performed using the PrimeScript® RT Master Mix Perfect Real Time kit (TAKARA, Japan). qPCR was conducted using SYBR Green Master Mix (Applied Biosystems, US) and a qPCR instrument (Applied Biosystems 7900HT Real-Time System; US). The relative levels of genes were analyzed by the 2^-ΔΔCt^ method and normalized to actin. The primer sequence information was forward primer 5′-GACATCACTGATGTCTCCAGAGC-3′ and reverse primer 5′-GAGCAGCACATGTT CTTCCTCATG-3′ for VDR, forward primer 5′-CAGCTGCTGTCCTATTCCGAATGT-3′ and reverse primer 5′-TGTCCAGTCCAAGATCTGCAGTCTC-3′ for β-catenin, forward primer 5′-A GTCATCAAGTGTGACCCGGTC-3′ and reverse primer 5′-TGGCGCAGGCTTGACTCCAGAA G-3′ for cyclin D1, forward primer 5′-ACATGCAGCTTTATCCAGGCTG-3′ and reverse primer 5′-TGCACGTTGGGAAGGAGCTTCTC-3′ for LEF-1.

### Western blot analysis

The proteins were extracted using RIPA protein extraction reagent (Beyotime, China) supplied with a cocktail (Roche, US). The lysis products were subjected to SDS–PAGE, transferred to polyvinylidene fluoride (PVDF) membranes, and blocked in 5% milk. The membranes were incubated with primary antibodies (1:1000; rabbit anti-VDR, rabbit anti-β-catenin, rabbit anti-cyclin D1, and anti-LEF-1) (Abcam, US) and secondary antibodies (1:5000; goat anti-rabbit IgG) (Abcam, US). Autoradiograms were quantified by densitometry using GAPDH as a control with ImageJ software.

### Cell apoptosis detection assay

SW480 cells infected with lenti-OE-VDR, lenti-shRNA-VDR and lenti-shRNA-VDR plus lenti-shRNA-β-catenin were trypsinized and washed twice with cold PBS. We detected SW480 cell apoptosis using the Annexin V-FITC/PI Cell Apoptosis Detection Kit (Transgen Biotech, China) following the manufacturer’s instructions on a flow cytometer (BD FACSCanto II, BD Biosciences, USA).

### Transwell invasion assay

We placed Transwell permeable supports in a 24-well plate. The upper side of the cell-permeable membranes was blocked with Matrigel (Solarbio, China). The cells were prepared for the cell apoptosis detection assay and transferred to blocked membranes. Culture medium with 20% FBS was added to the wells of the 24-well plate. The Transwell inserts were removed, and then the membranes were washed with PBS twice and the cells were fixed with methanol after 24–48 h of conventional cultivation. The membranes were stained with 1% crystal violet after gently wiping off the upper layer of nonmigrated cells. We counted the cells in the lower layer of the membranes under a microscope using 5 random fields of view.

### LUC reporter assay

Forty-eight hours after transfection, the cells were lysed and subjected to the LUC reporter assay. The ratio of the activities of the two luciferases (firefly luciferase and Renilla luciferase) was used as the relative luciferase activity. The relative LUC activity was detected following the instructions of the Dual-Luciferase Reporter Assay Kit (Promega, US) with the Modulus Single Tube Fluorometer (Turner Biosystems, US).

### ChIP assay

ChIP was conducted using the SimpleChip Plus Enzymatic Chromatin IP Kit (Cell Signaling Technology, US) in SW480 cells following the manufacturer’s instructions. Immunoprecipitation was performed using a specific VDR antibody (Abcam, US) compared with the control IgG. Twenty percent of each sample was reserved as Input. The relative immunoprecipitated DNA was normalized to the results of IgG controls and quantified to the respective inputs. Three biological repeats of each group were conducted.

### Co-IP assay

The Protein A/G Immunoprecipitation Kit (Invitrogen, US) was used to enrich the VDR and β-catenin proteins. Cells expressing Myc-VDR, Myc-IRF9 (IRF9 was not related to Wnt/β-catenin pathway) and β-catenin were lysed, and 5 mg of each protein was used for immunoprecipitation with 100 μg of IgA and Myc antibodies (Abcam, China). The immunoprecipitation of the interacting protein β-catenin was controlled by Myc-VDR and Myc-IRF9 with empty pcDNA3.1 respectively and Myc-IRF9 with β-catenin. Western blotting analysis of the input was used to determine the initial protein levels of VDR and β-catenin in each group.

### GST pull-down analysis

The fusion proteins of GST-β-catenin were prepared as described previously [31]. Approximately 100 μg of GST-β-catenin fusion protein was incubated with 50 μL of glutathione agarose (Yeasen, China) at 4 °C for 1 h with gentle rocking. Approximately 100 μg of Myc-VDR fusion protein was added to the immobilized GST-β-catenin solutions and incubated together at 4 °C overnight with gentle rotation. PBS solutions with 10 mM glutathione (pH 8.0) were used to elute the bound protein Myc-VDR and analyzed by Western blotting. Beta-Actin was employed as a loading control.

### VDR immunohistochemical score (IS)

The immunohistochemical staining results of VDR in the tissues were considered positive with brown particles in the cytoplasm/nucleus. Five fields of view were randomly selected for each slice under the microscope (× 200), and the percentage of positive cells was calculated. The final score was calculated by the product of ‘staining intensity × percentage of positive cells’. An IS value greater than 5 indicated high expression, and an IS value less than or equal to 4 indicated low expression.

### Statistical analysis

Statistical analysis was conducted using GraphPad Prism 5.0 (GraphPad Inc., US) and SPSS 22.0 (IBM Corporation, US). Student’s t-test and one-way ANOVA were used to analyze the differences between distinctive groups. All the data were presented as means ± SD, and *P* < 0.05 was considered statistically significant.

## Results

### VDR expression is closely related to the development and prognosis of CRC patients

**Fig. 1:**
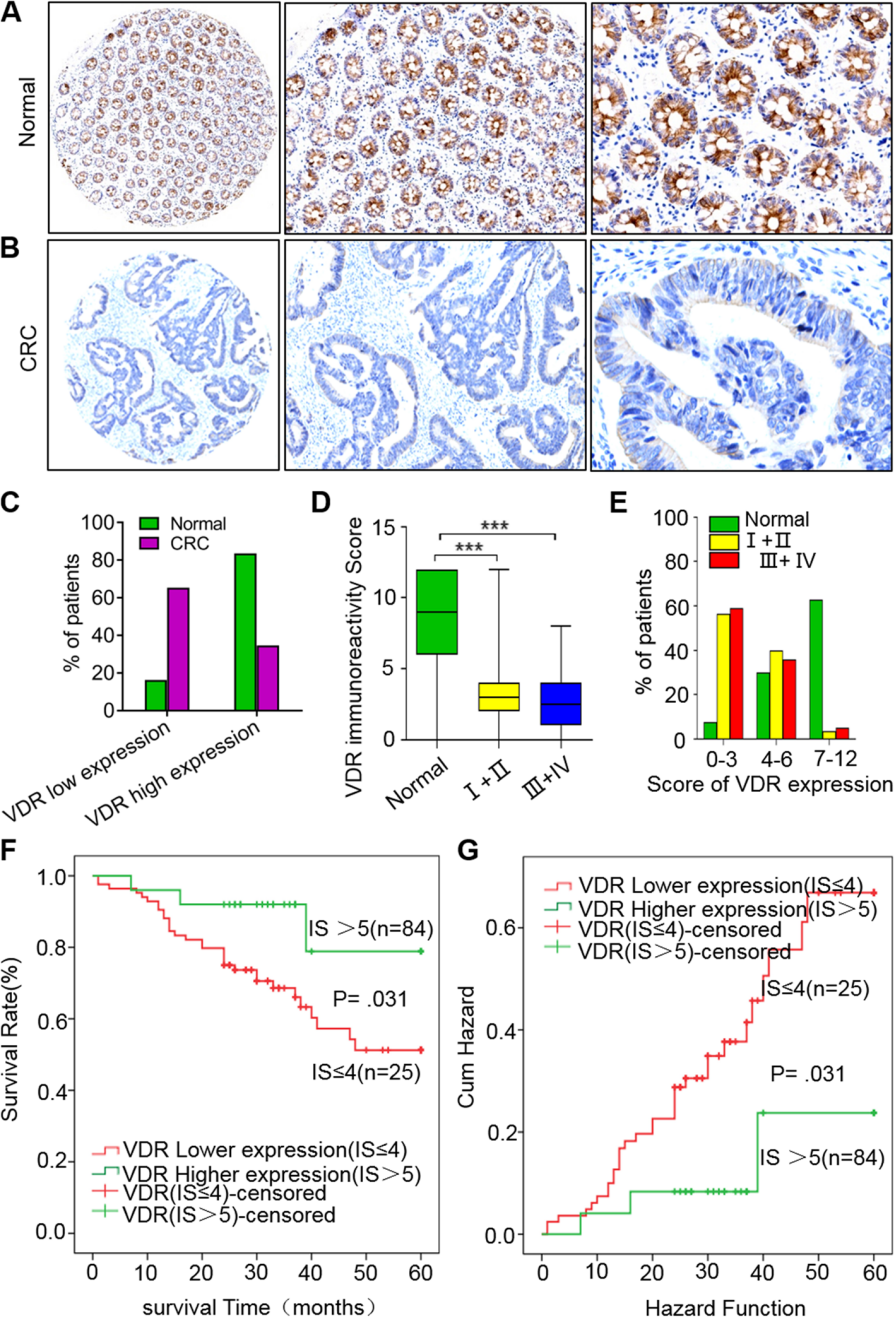
VDR expression is closely related to the development and prognosis of CRC. **a** and **b** VDR expression in normal and CRC tissues. **c** Percentage of patients with low and high VDR expressions respectively in normal and CRC tissues. **d** VDR immunoreactivity scores in normal tissues and stage I + II and III + IV CRC tissues. **e** Percentage of patients with different VDR expression scores in normal tissues and stage I + II and III + IV CRC tissues. **f** Survival rates of patients with different VDR immunohistochemical scores (IS). **g** Curn hazard analysis of the patients with different VDR ISs. The VDR immunoreactivity scores were calculated according to the percentage of normal and CRC tissues with high VDR expression. The data are represented as means ± SD, *n* ≥ 100，***P < *0.001*

To determine the role of VDR in CRC, we collected patient tissues from the Department of Pathology, People’s Hospital of Suzhou High-tech Zone; 188 samples were from patients were diagnosed with CRC tissues, and 134 were from healthy subjects. Immunohistochemistry showed that in normal and CRC colorectal tissues, the VDR proteins were mainly located in the cytoplasm (Fig. 1a, b). Further analysis showed that the high expression rates of VDR protein in normal and CRC tissues were 83.6% (112/134) and 34.6% (65/188), respectively (Fig. 1c).

Furthermore, the high expression rate of VDR in CRC tissues without lymph node metastasis was 42.2% (43/102), significantly higher than that of cases with lymph node metastasis (25.6% (22/86); *P* = 0.017; Table 1). The high expression rates of VDR in cases with invasion depths T1-T2 and T3-T4 were 43.6% (34/78) and 28.2% (31/110), respectively, revealing significant differences (*P* = 0.029; Table 1). The rate was 44.5% (49/110) in stage I + II CRC tissues, significantly higher than the 20.5% (16/78) in stage III + IV tissues (*P* = 0.001; Table 1). Thus, the VDR immunoreactivity scores of I + II and III + IV CRC tissues were significantly lower than those of normal tissues (Fig. 1d). Additionally, a higher percentage of patients with normal tissues revealed higher VDR expression. However, the opposite was true in I + II and III + IV CRC tissues, which showed a lower percentage of patients with higher VDR expression (Fig. 1e). Additionally, statistical analysis indicated that VDR expression was not significantly correlated with sex, age, tumour location, tumour size, general type, or degree of differentiation of CRC patients (Table 1).

**Table 1:** Correlations between VDR expression of CRC and clinicopathological factors

Variables	VDR expression			***P***-value
	Total Cases (%)	Low No. (%)	High No. (%)	
	(***n*** = 188)			
**Gender**				0.458
**Male**	111(59.04)	75(67.6)	36(32.4)	
**Female**	77(40.96)	48(62.3)	29(37.7)	
**Age (yrs)**				0.365
**≤60**	54(28.72)	38(70.40)	16(29.6)	
**>60**	134(71.28)	85(63.40)	49(36.6)	
**Tumor location**				0.645
**Colon**	91(48.40)	61(67.0)	30(33.00)	
**Rectum**	97(51.60)	62(63.9)	35(36.10)	
**Size (cm)**				0.206
**≤5 cm**	98(52.13)	60(61.20)	38(38.80)	
**>5 cm**	90(47.87)	63(70.00)	27(30.00)	
**General type**				0.183
**Bulge type**	61(32.45)	36(59.00)	25(41.00)	
**Ulcer type**	114(60.64)	76(66.70)	38(33.30)	
**Mushroom type**	13(6.91)	11(84.60)	2(15.40)	
**Differentiation**				0.093
**High**	84(44.68)	48(57.10)	36(42.90)	
**Middle**	76(40.43)	54(71.10)	22(28.90)	
**Low**	28(14,89)	21(75.00)	7(25.00)	
**Invasion depth**				**0.029**
**T1-T2**	78(41.49)	44(56.40)	34(43.60)	
**T3-T4**	110(58.50)	79(71.80)	31(28.20)	
**Lymph node metastasis**				**0.017**
**N0**	102(54.26)	59(57.80)	43(42.20)	
**N1-N3**	86(45.74)	64(74.40)	22(25.60)	
**TNM Stage**				**0.001**
**I + II**	110(58.50)	61(55.5)	20(44.50)	
**III + IV**	78(41.50)	62(79.50)	5(20.50)	

We conducted regular follow-ups on 109 CRC patients. The Kaplan–Meier method was used to analyze the relationship between VDR expression and the postoperative survival rate of CRC patients. With the decrease in VDR expression, the postoperative survival time of CRC patients showed a gradually shortening trend (Fig. 1f). The difference in the postoperative survival rate and Cum Hazard analysis of CRC patients with high and low VDR expression were statistically significant (Fig. 1f, g).

### Construction of lentivirus-delivered VDR and β-catenin silencing and VDR overexpression constructs in SW480 cells

To clarify the molecular mechanism by which VDR regulates the development of CRC, we designed lentivirus-delivered VDR silencing and overexpression constructs (Figs. S1 and S2). VDR expression in SW480 cells transfected with the three shRNAs showed that VDR shRNA-2 exhibited relatively high silencing efficiency (Fig. S1a, b). Additionally, VDR expression in the lentivirus-delivered FG-VDR-eGFP increased (Fig. S2). Moreover, β-catenin expression in SW480 cells transfected with the three shRNAs showed that β-catenin shRNA-3 exhibited relatively high silencing efficiency (Fig. S3). Thus, VDR shRNA-2 and β-catenin shRNA-3 were applied in subsequent experiments, respectively.

### VDR and β-catenin regulate the invasion and apoptosis of SW480 cells

**Fig. 2:**
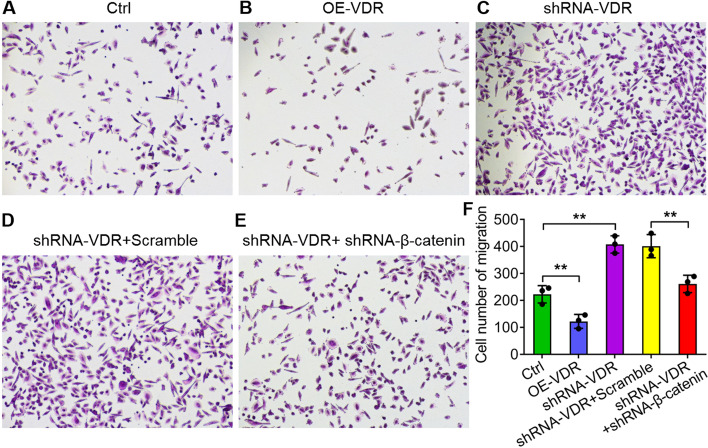
Detection of cell invasion ability by Transwell experiments. **a-c** Invasion of SW480 cells using lentivirus-delivered VDR overexpression (**b**) and VDR interference (**c**) compared with the controls (**a**). **d** and **e** Invasion of SW480 cells with VDR and β-catenin interference simultaneously (**e**) compared with the VDR interference plus scramble groups (**d**). **f** Quantitative analysis of the migration number of SW480 cells in **a** to **e**. The data are presented as means ± SD, *n* ≥ 3, **P < *0.01*

**Fig. 3:**
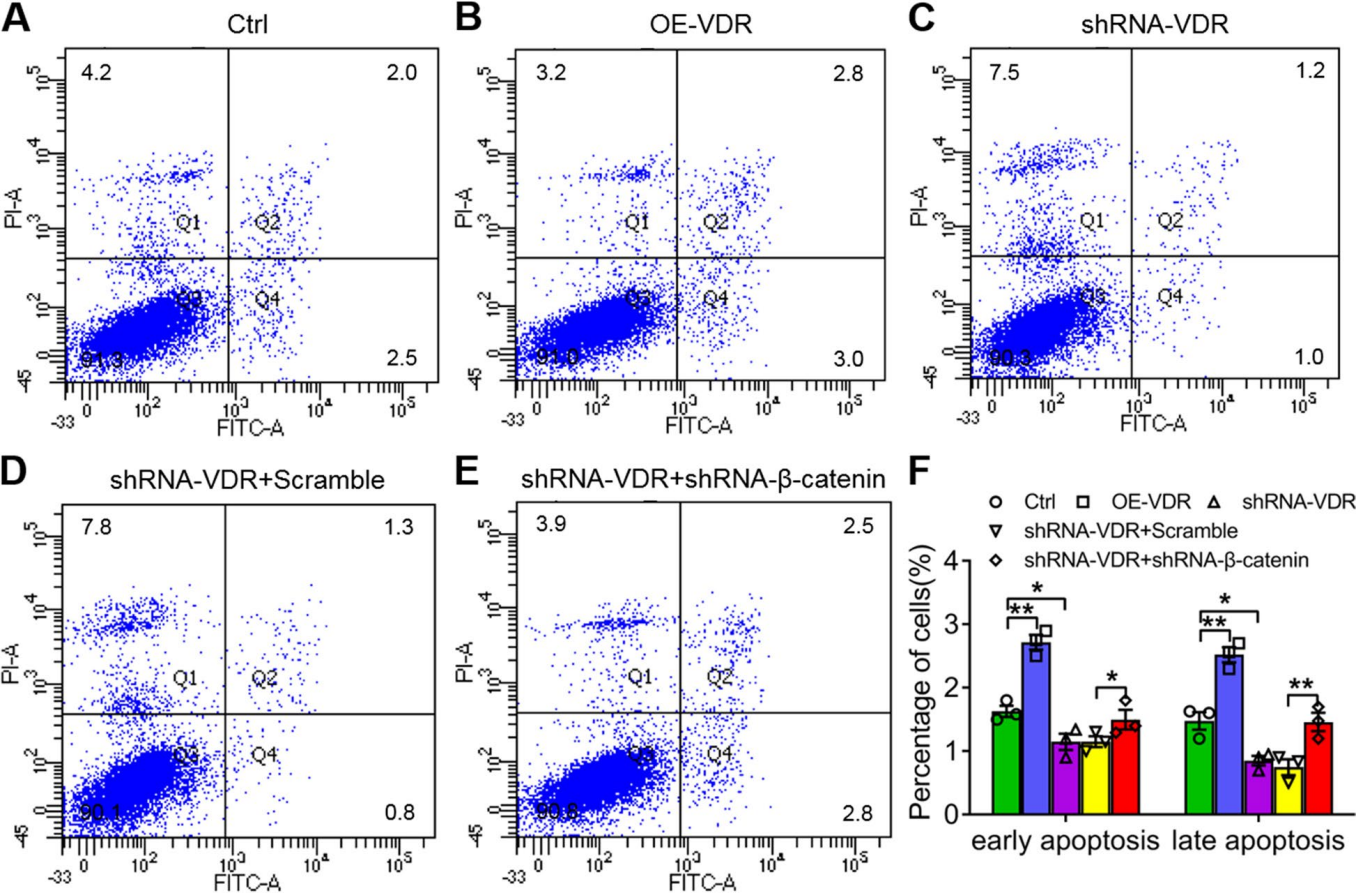
Detection of apoptosis of SW480 cells by flow cytometry analysis. **a-c** Early and late apoptosis of SW480 cells with lentivirus-delivered VDR overexpression (**b**) and VDR interference (**c**) compared with the controls (**a**). **d** and **e** Early and late apoptosis of SW480 cells with VDR and β-catenin interference simultaneously (**e**) compared with the VDR interference plus scramble groups (**d**). **f** Quantitative analysis of the percentage of early and late apoptosis of SW480 cells in **a** to **e**. The data are presented as means ± SD, *n* ≥ 3, *P < *0.05*, **P < *0.01*

Tumour invasion and metastasis play key roles in cancer progression. According to the results from the Transwell experiment, the invasion ability of SW480 cells was reduced significantly with lentivirus-delivered VDR overexpression compared with that of the controls (**P < *0.01*; Fig. 2a, b, f). However, the invasion ability visibly and significantly increased with lentivirus-delivered VDR interference (**P < *0.01*; Fig. 2a, c, f). Resistance to apoptosis is an important hallmark in human cancers. According to the results of flow cytometry analysis, both early and late apoptosis significantly increased with VDR overexpression (**P < *0.01*; Fig. 3a, b, f), but they both decreased significantly in the shRNA-VDR groups compared with the controls in SW480 cells (**P < *0.01*; Fig. 3a, c, f).

To identify the relationship between VDR and Wnt/β-catenin signalling in CRC, we silenced β-catenin with shRNA and detected invasion and apoptosis in SW480 cells. The invasion ability significantly decreased with the simultaneous interference of VDR and β-catenin compared with the shRNA VDR + scramble groups (**P < *0.01*; Fig. 2d-f). By contrast, the early and late apoptosis of SW480 cells increased significantly with both VDR and β-catenin interference (*P < *0.05*; **P < *0.01*; Fig. 3d-e). These results indicated that both VDR and β-catenin were involved in CRC cell activity.

### VDR regulates the expression of β-catenin, cyclin D1 and LEF-1

**Fig. 4:**
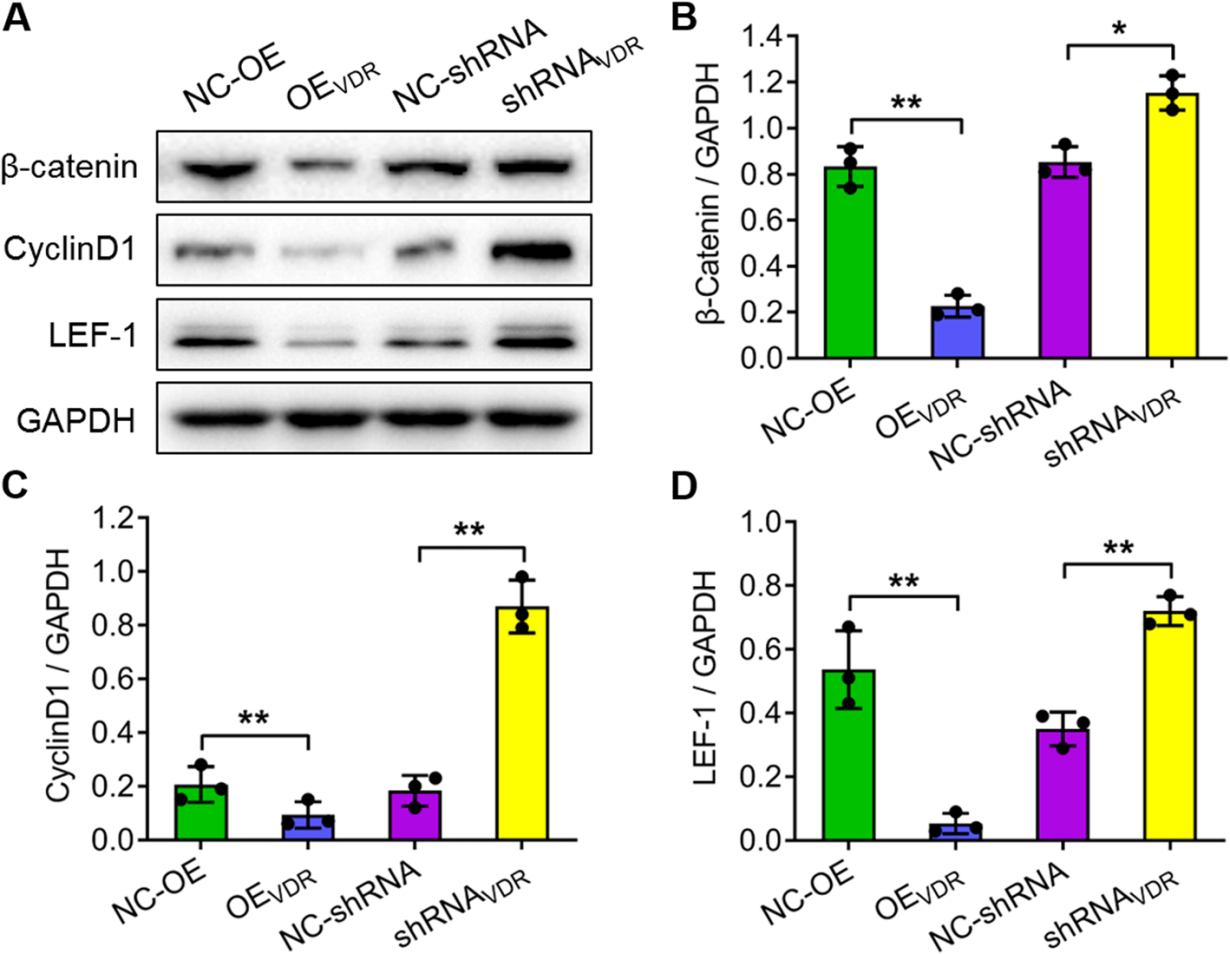
Protein expression of β-catenin, cyclin D1 and LEF-1 under VDR overexpression and interference conditions in SW480 cells. **a** Western blotting analysis revealed the protein expression of β-catenin, cyclin D1 and LEF-1 under VDR overexpression and interference conditions normalized to GAPDH. **b-d** Quantitative analysis of β-catenin, cyclin D1 and LEF-1 protein expression in **a**. The data are presented as means ± SD, n ≥ 3, *P < *0.05*, **P < *0.01*

**Fig. 5:**
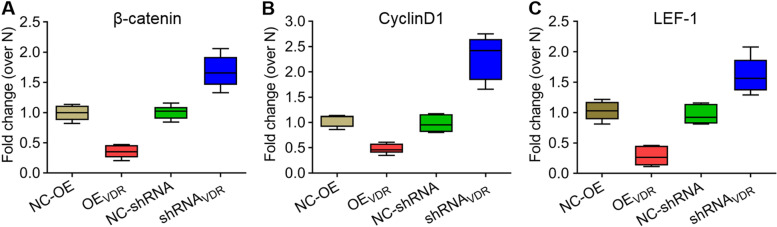
mRNA expression of β-catenin (**a**), cyclin D1 (**b**) and LEF-1 (**c**) under VDR overexpression and interference conditions in SW480 cells. The data are represented as means ± SD, n ≥ 3, *P < *0.05*, **P < *0.01*

To further determine the impact of VDR on the expression of genes in the Wnt/β-catenin signalling pathway, we detected the protein and mRNA expression levels of β-catenin, cyclin D1 and LEF-1 under VDR overexpression and interference conditions in SW480 cells. Western blotting revealed that the expression of the three genes significantly decreased in VDR-overexpressing cells but increased significantly in VDR-silenced cells compared with the controls (*P < *0.05*; **P < *0.01*; Fig. 4). The mRNA expression of these genes from qRT–PCR results confirmed the Western blotting analysis (Fig. 5).

### VDR interacts with β-catenin at the protein level, and VDR overexpression prevents the nuclear accumulation of β-catenin

**Fig. 6:**
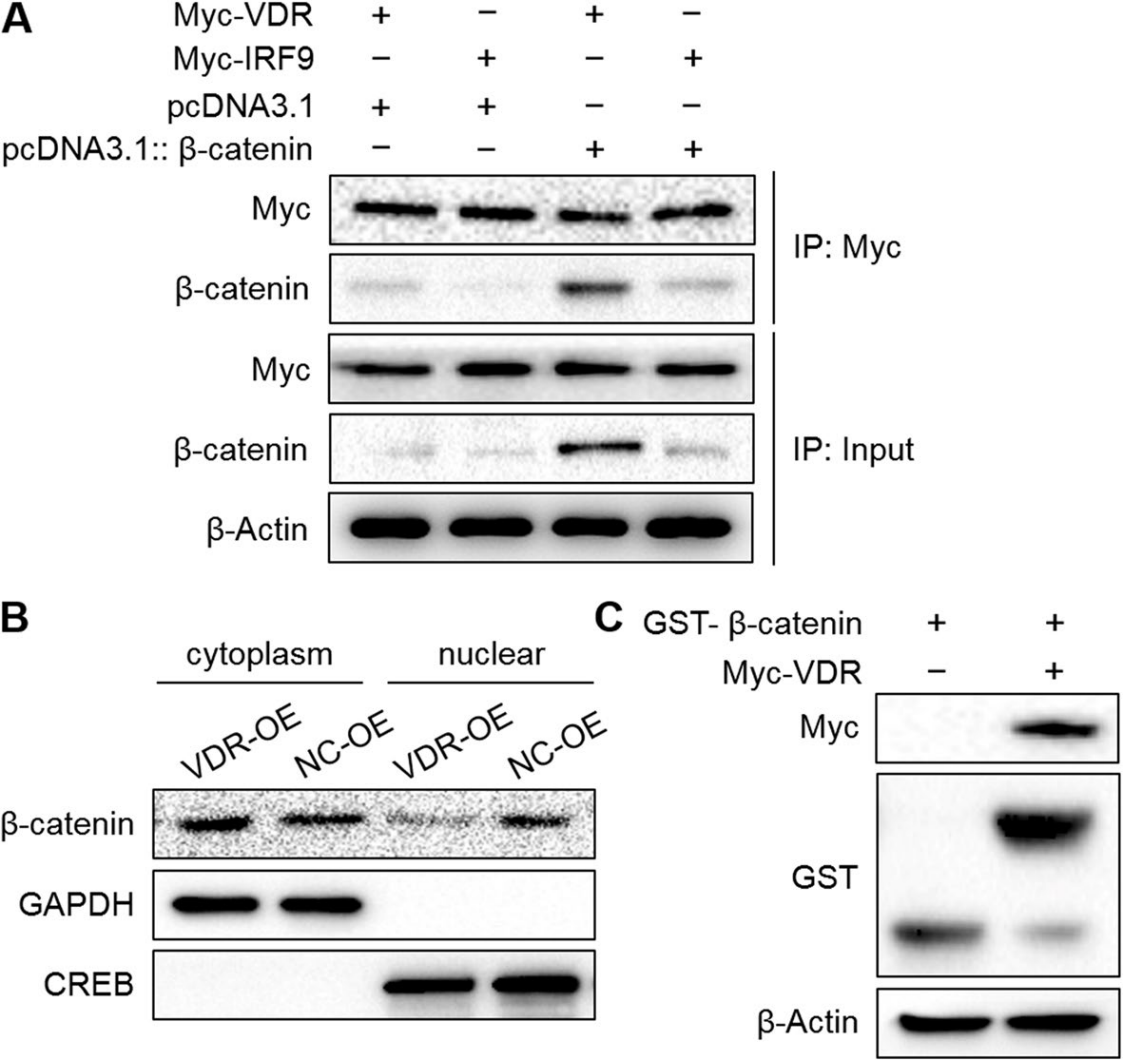
VDR interacts with β-catenin at the protein level, and VDR overexpression promotes the nuclear accumulation of β-catenin. **a** The Co-IP assay showed that the Myc antibody efficiently immunoprecipitated the β-catenin proteins controlled by the input. The initial biomasses were normalized to beta-Actin. **b** Western blotting analysis showed the accumulation of β-catenin in the cytoplasm and nucleus with VDR overexpression. GAPDH and CREB were used as protein controls. **c** The GST pull-down assay showed that the GST-β-catenin fusion proteins could pull down Myc-VDR efficiently. Beta-Actin was used as the protein control

To further identify the molecular relationship between VDR and β-catenin, we performed Co-IP and GST pull-down assays using Myc-fused VDR and GST-fused β-catenin with recombinant DNA techniques (Fig. 6). According to the Co-IP assay results, the Myc antibody efficiently immunoprecipitated the β-catenin proteins (Fig. 6a). The GST-β-catenin fusion proteins efficiently pulled down the fused Myc-VDR proteins (Fig. 6c). Additionally, we isolated the cytoplasm and nucleus in VDR-overexpressing SW480 cells. Western blotting showed that the β-catenin proteins were translocated to the cytoplasm (Fig. 6b). These results indicated that VDR interacted with β-catenin at the protein level and VDR overexpression prevented the nuclear accumulation of β-catenin.

### VDR directly activates LEF-1 expression

**Fig. 7:**
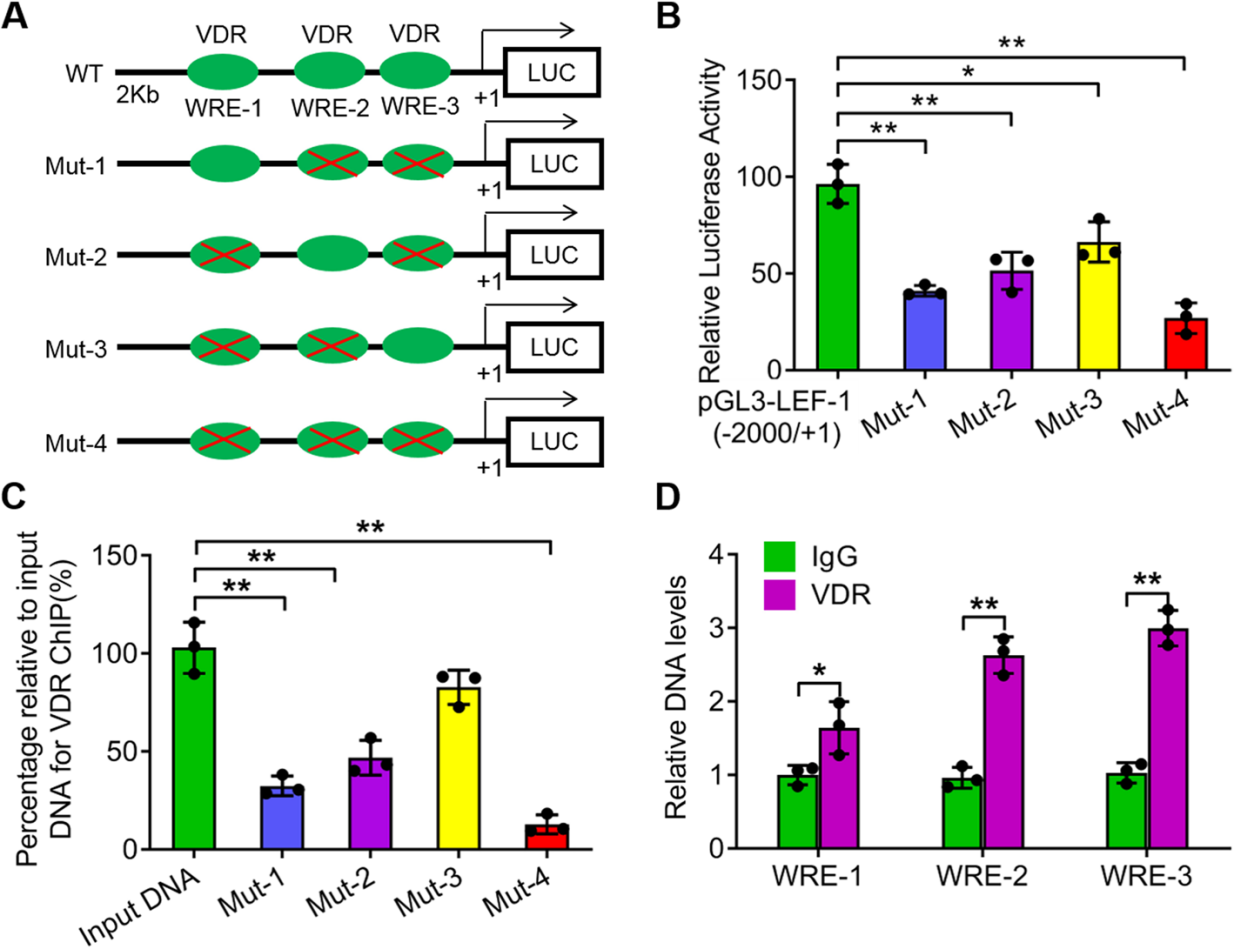
VDR directly activates LEF-1 expression in SW480 cells. **a** The diagram represents the LUC reporter construction containing three putative VDR binding sites (WRE-1, WRE-2 and WRE-3) in the LEF-1 promoter region (2000 bp upstream of the ATG) and four mutated LEF-1 promoters with only one WRE binding site or none. **b** The relative LUC activity of the constructs in A from the dual LUC reporter assay. **c** The percentage relative to input DNA for VDR ChIP was quantified. **d** The relative DNA levels of the three WRE binding sites from VDR ChIP were quantified and controlled by IgG. The data are represented as means ± SD, n ≥ 3, *P < *0.05*, **P < *0.01*

β-catenin plays key roles in the activity of the TCF/LEF protein complex. VDR is a nuclear transcription factor that interacts with the β-catenin protein in CRC cells. We speculated that VDR might activate the expression of TCF/LEF. We performed bioinformatics analysis of the LEF-1 promoter regions, revealing that the LEF-1 promoter contained three putative binding sites for VDR (Fig. 7a). To better understand the binding efficiency contributed by the three putative WREs, we mutated the WREs in order (Fig. 7a). The relative LUC activity quantified by the dual LUC reporter assay showed that VDR proteins could bind to the LEF-1 promoter and trigger the expression of the LUC reporter (*P < *0.05*; **P < *0.01*; Fig. 7b). Compared with the construct containing the 2000 bp LEF-1 promoter sequence, the LUC activity of the four mutated constructs was significantly decreased (*P < *0.05*; **P < *0.01*; Fig. 7b). The three WREs were essential for LEF-1 activation because the LUC activities of Mut-1 to Mut-3 were relatively higher than those of Mut-4 (Fig. 7b). Additionally, we performed ChIP-PCR to further confirm the results of the dual LUC reporter assay. The VDR ChIP assay showed that the relative DNA levels of the fragments containing the three WREs immunoprecipitated by VDR proteins were significantly higher than those in the IgG controls (*P < *0.05*; **P < *0.01*; Fig. 7d). The percentage of DNA for VDR ChIP relative to the input confirmed this result and revealed that all three WREs contributed to the binding for VDR (**P < *0.01*; Fig. 7c).

### VDR overexpression significantly reduces tumour growth in nude mice by inhibiting the expression of β-catenin, cyclin D1 and LEF-1

**Fig. 8:**
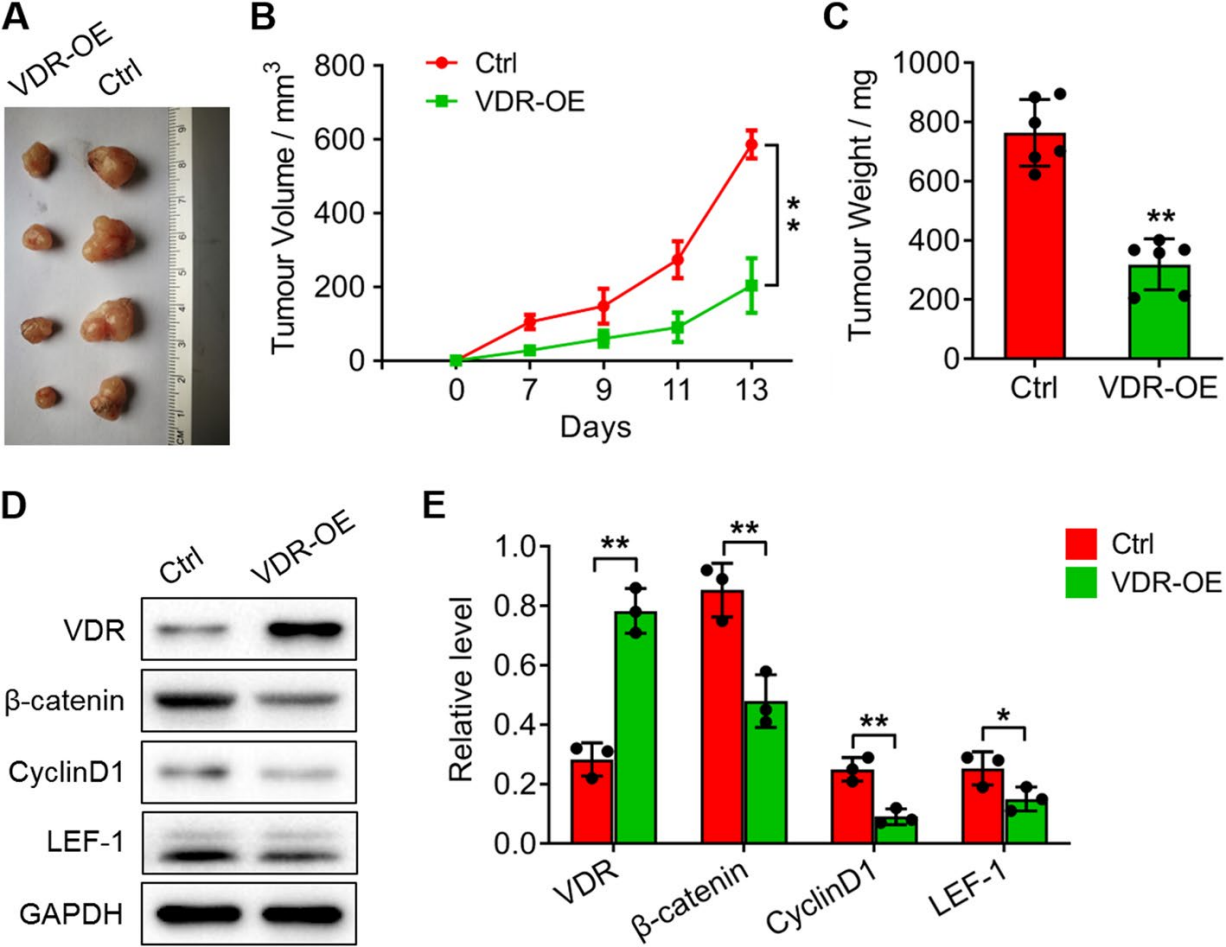
VDR overexpression suppresses tumour growth and inhibits the expression of β-catenin, cyclin D1 and LEF-1. **a** Tumours in the nude mice injected with VDR-overexpressing SW480 cells compared with the controls. **b** Quantitative analysis of the tumour volume within 13 days. **c** Quantitative analysis of tumour weight in A. **d** Western blotting analysis showed the protein levels of β-catenin, cyclin D1 and LEF-1 in the mice of A. **e** Quantitative analysis of the relative protein levels of **d**. The data are presented as means ± SD, n ≥ 3, *P < *0.05*, **P < *0.01*

The above results suggested that VDR regulated invasion and apoptosis in CRC cells. To support these findings, we injected VDR-overexpressing SW480 cells into nude mice (Fig. 8). Thirteen days later, the tumour volume and weight from the VDR-overexpressing mice were significantly reduced compared with those of the controls (**P < *0.01*; Fig. 8a-c). Additionally, the protein levels of β-catenin, cyclin D1 and LEF-1 were significantly decreased compared with the increase in VDR expression compared with the controls (*P < *0.05*; **P < *0.01*; Fig. 8d, e).

## Discussion

CRC, the 3rd most common cancer worldwide, has been a public health issue threatening human lives. Approximately 1.8 million CRC patients were diagnosed worldwide in 2018 [32]. With the increasing incidence of this cancer, CRC is one of the leading causes of death worldwide [33, 34]. However, the underlying mechanisms of CRC initiation and development remain unclear, indicating the need to improve the understanding of the molecular basis of CRC development to identify efficient early diagnostic markers and reliable therapeutic strategies [35]. VDR expression was indicated to closely associate with CRC progression and serum VDR reduction was showed previously in CRC tissues compared to the adjacent tissues [36–38]. In our study, according to the results from 188 CRC patients and 134 normal cases, only 34.6% of the CRC cases exhibited relatively high VDR expression, but 83.6% of the normal cases showed high VDR expression. Additionally, the difference was statistically significant. These findings confirmed that VDR expression is closely related to the development and prognosis of CRC patients. High VDR expression may be beneficial to suppress CRC development. Further findings revealed that high VDR expression was significantly correlated with CRC progression and that higher VDR levels presented a greater probability in CRC tissues without lymph node metastasis/with lower invasion depth/with early TNM stage. Furthermore, the results from regular follow-ups on 109 CRC patients showed that the postoperative survival times of CRC patients gradually shortened with decreasing VDR expression. These findings confirmed that higher VDR expression was vital to inhibit CRC progression in patients.

Cancer metastasis is the leading cause of cancer-related death [39]. Considering that cell migration and invasion into surrounding tissues are crucial steps for cancer metastasis, we detected the impact of VDR expression on CRC cell migration and invasion. Overexpression of VDR in SW480 cells significantly inhibited invasion. Additionally, VDR overexpression resulted in an increase in early and late cell apoptosis. Cell apoptosis is widely known as programmed cell death, which is crucial for animal development [40]. Apoptotic cell death inhibits oncogenesis, including metastasis [41]. Therefore, cell death plays major roles in anticancer therapies. Taken together, these results demonstrated that VDR is essential for CRC cell invasion and apoptosis and that high VDR levels are required to suppress CRC cell invasion and increase apoptosis to inhibit CRC development, findings that are consistent with those in CRC patients.

Accumulating evidence indicates that Wnt/β-catenin signalling plays essential roles in embryo development [26, 42]. Wnt/β-catenin signalling regulates several cellular processes, such as proliferation, differentiation, motility and potency maintenance. Aberrant Wnt signalling contributes to many cancer types. Deregulation of this pathway is always accompanied by carcinogenesis and is found in almost all CRC patients [26]. Among the alterations, mutations in β-catenin genes often occur. VDR is a crucial modulator of nuclear β-catenin activities, and VDR regulates nuclear β-catenin levels in CRC cells and therefore attenuates the impact of the activation the Wnt/β-catenin pathway [43, 44]. In the present study, VDR overexpression decreased the mRNA and protein levels of β-catenin in vitro and in vivo, a finding that is consistent with previous findings in murine melanoma cells that elevated vitamin D-VDR levels inhibit Wnt/β-catenin signalling [45]. Silencing of β-catenin and VDR together in SW480 cells reduced cell invasion and increased apoptosis significantly compared with those in the VDR shRNA-only groups, partially rescuing the impact of VDR interference. Additionally, VDR overexpression prevented β-catenin accumulation in the nucleus in vitro and suppressed tumour development in vivo. Wnt/β-catenin signalling was confirmed to be activated by β-catenin nuclear accumulation in approximately 90% of CRC tumours [26]. Taken together, these results suggested that β-catenin functions downstream of and is inhibited by VDR signalling to block CRC progression. In the present study, the expression of cyclin D1 and LEF-1 was reduced by VDR overexpression in vitro and in vivo. VDR activates the expression of LEF-1 through binding with β-catenin. Due to VDR overexpression significantly reduced the expression of β-catenin and resulted in β-catenin exporting from the nucleus and accumulating in the cytoplasm, it might inhibit Wnt/β-catenin signalling and subsequently weaken the binding of VDR to the LEF-1 promoter, thus the expression of LEF-1 revealed decreased with VDR expression. Taking together with the evidence that cyclin D1 is a direct target of the β-catenin/LEF-1 complex and elevated cyclin D1 expression has been implicated in the pathogenesis of many diseases by stimulating cell proliferation [46, 47], we demonstrated that VDR plays a crucial role in the process of β-catenin entering the nucleus and regulating the transcription of Wnt target genes, VDR levels may contribute to controlling CRC tumour development by inhibiting Wnt/β-catenin signalling, and VDR expression maintenance is closely associated with fewer metastatic CRC diseases in humans.

## Conclusions

In summary, this study verifies that VDR plays key roles in human CRC progression by regulating the Wnt/β-catenin signalling pathway to control cancer cell invasion and apoptosis based on molecular biological and clinical analysis and provides new evidence to explore VDR biomarkers or agonists for CRC patient diagnosis and treatment. The molecular mechanism underlying the VDR/Wnt/β-catenin signalling pathway should be complicated and warrants further study.

## Supplementary Information

**Additional file 1: Fig. S1.** VDR expression of lentivirus-delivered shRNAs in SW480 cells. (a) eGFP signals from the three different lentivirus-delivered VDR shRNAs. Titer = 7.2 × 10^6^ PFU/mL. (b) Western blotting analysis of VDR expression in the three different shRNAs normalized to GAPDH. **Fig. S2.** VDR expression in SW480 cells overexpressing lentivirus-delivered VDR. (a-e) eGFP signals in the lentivirus-delivered OE-VDR. Titer = 8.9 × 10^7^ PFU/mL. (f) Western blotting analysis of VDR expression in OE-VDR normalized to GAPDH. **Fig. S3.** β-catenin expression of lentivirus-delivered shRNAs in SW480 cells. (a) eGFP signals from the three different lentivirus-delivered β-catenin shRNAs. Titer = 7.8 × 10^6^ PFU/mL. (b) Western blotting analysis of β-catenin expression in the three different shRNAs normalized to GAPDH.

**Additional file 2.**

## Data Availability

The datasets used and/or analysed during the current study are available from the corresponding author on reasonable request.

## Abbreviations

CRC: Colorectal cancer
VDR: vitamin D receptor
LEF: lymphoid enhancer factor
Co-IP: Co-immunoprecipitation
GST: glutathione-S-transferase
ChIP: chromatin immunoprecipitation
WHO: World Health Organization
APC: adenomatous polyposis coli
GSK3beta: glycogen synthase kinase-3beta
CK: casein kinase
TCF: T-cell factor
qRT–PCR: Quantitative real-time PCR
LUC: Dual luciferase
