# Supplementary material for: Vitamin D receptor prevents tumour development by regulating the Wnt/β-catenin signalling pathway in human colorectal cancer
Source: BMC Cancer. 2023 Apr 12;23:336. doi: 10.1186/s12885-023-10690-z (PMC10091620; doi:10.1186/s12885-023-10690-z)
Supplement: Supplementary file 1 — Additional file 1: Fig. S1. VDR expression of lentivirus-delivered shRNAs in SW480 cells. (a) eGFP signals from the three different lentivirus-delivered VDR shRNAs. Titer = 7.2 × 10^6^ PFU/mL. (b) Western blotting analysis of VDR expression in the three different shRNAs normalized to GAPDH. Fig. S2. VDR expression in SW480 cells overexpressing lentivirus-delivered VDR. (a-e) eGFP signals in the lentivirus-delivered OE-VDR. Titer = 8.9 × 10^7^ PFU/mL. (f) Western blotting analysis of VDR expression in OE-VDR normalized to GAPDH. Fig. S3. β-catenin expression of lentivirus-delivered shRNAs in SW480 cells. (a) eGFP signals from the three different lentivirus-delivered β-catenin shRNAs. Titer = 7.8 × 10^6^ PFU/mL. (b) Western blotting analysis of β-catenin expression in the three different shRNAs normalized to GAPDH. [file 12885_2023_10690_MOESM1_ESM.docx]

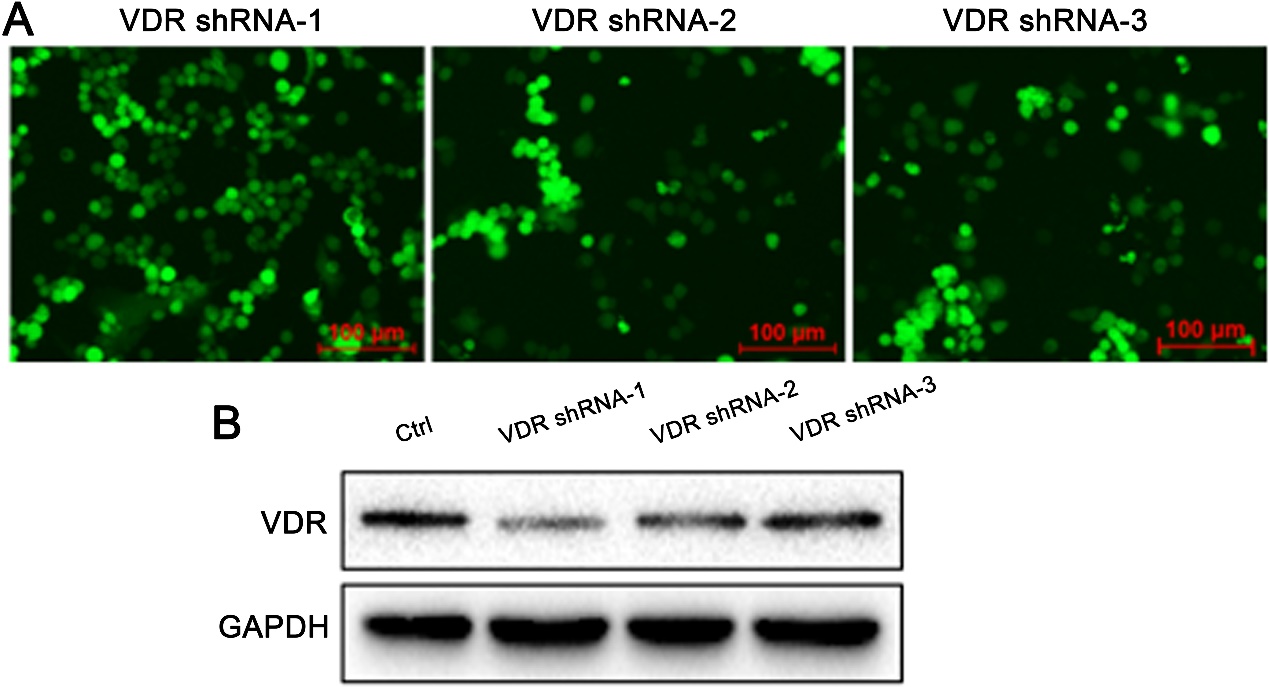


**Figure S1.** VDR expression of lentivirus-delivered shRNAs in SW480 cells. (**a**) eGFP signals from the three different lentivirus-delivered VDR shRNAs. Titer = 7.2×10^6^ PFU/mL. (**b**) Western blotting analysis of VDR expression in the three different shRNAs normalized to GAPDH.


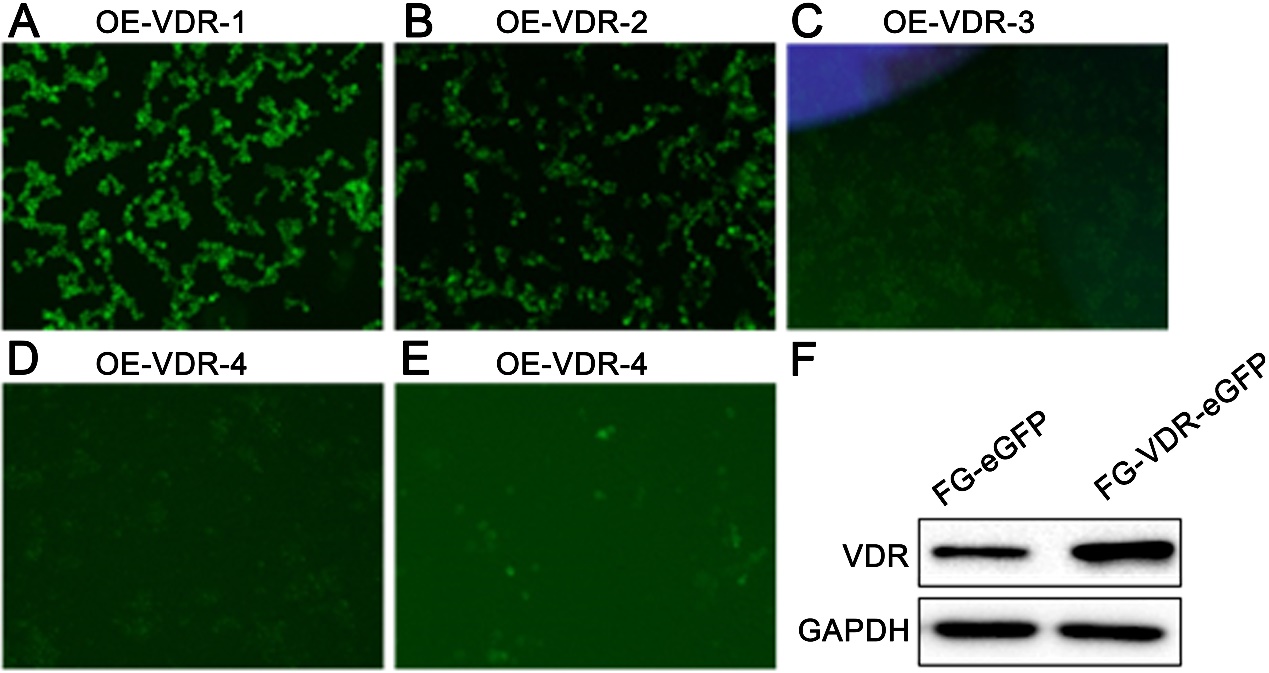


**Figure S2.** VDR expression in SW480 cells overexpressing lentivirus-delivered VDR. (**a-e**) eGFP signals in the lentivirus-delivered OE-VDR. Titer = 8.9×10^7^ PFU/mL. (**f**) Western blotting analysis of VDR expression in OE-VDR normalized to GAPDH.


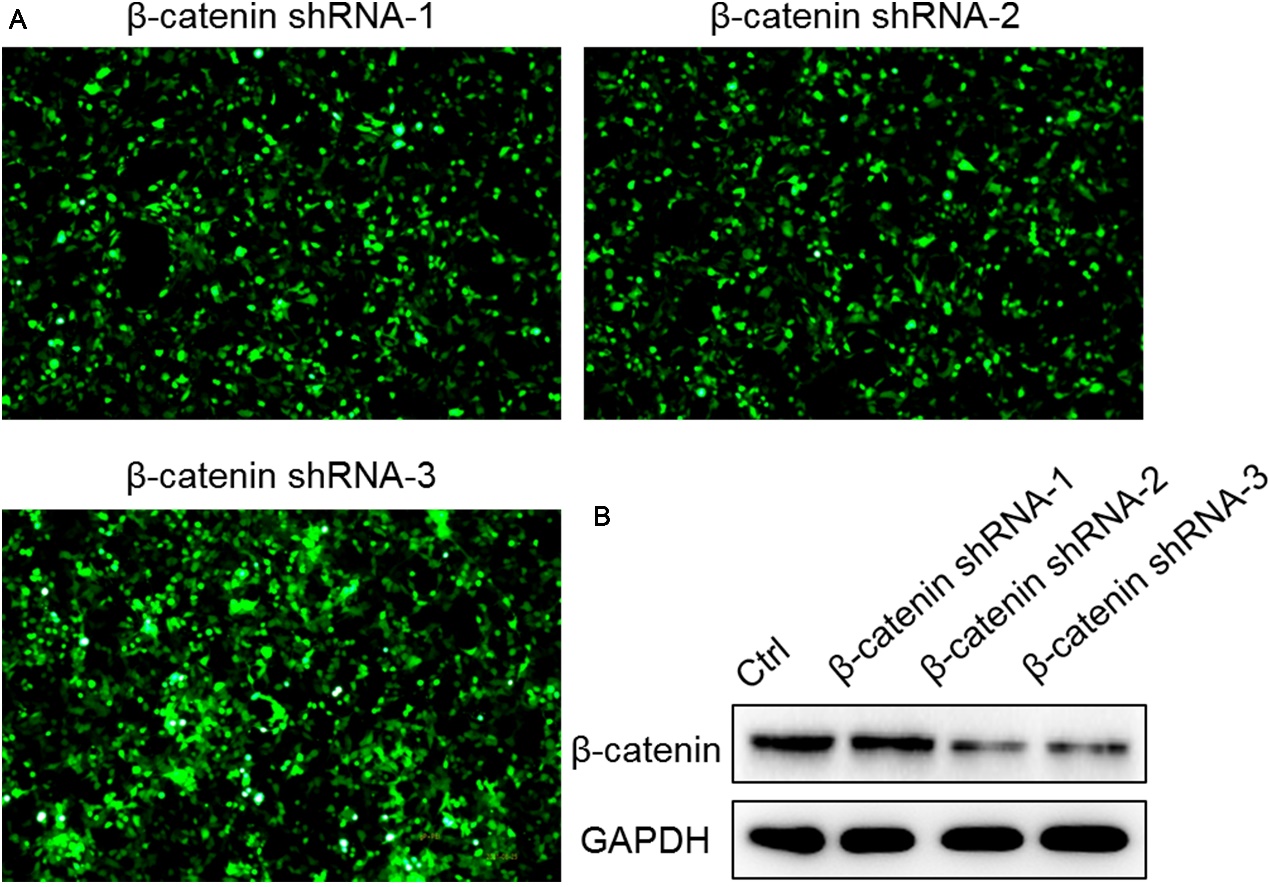


**Figure S3.** β-catenin expression of lentivirus-delivered shRNAs in SW480 cells. (**a**) eGFP signals from the three different lentivirus-delivered β-catenin shRNAs. Titer = 7.8×10^6^ PFU/mL. (**b**) Western blotting analysis of β-catenin expression in the three different shRNAs normalized to GAPDH.
