# Supplementary material for: Vitamin D receptor prevents tumour development by regulating the Wnt/β-catenin signalling pathway in human colorectal cancer
Source: BMC Cancer. 2023 Apr 12;23:336. doi: 10.1186/s12885-023-10690-z (PMC10091620; doi:10.1186/s12885-023-10690-z)
Supplement: Supplementary file 2 — Additional file 2. [file 12885_2023_10690_MOESM2_ESM.docx]

Supplementary fig. 1


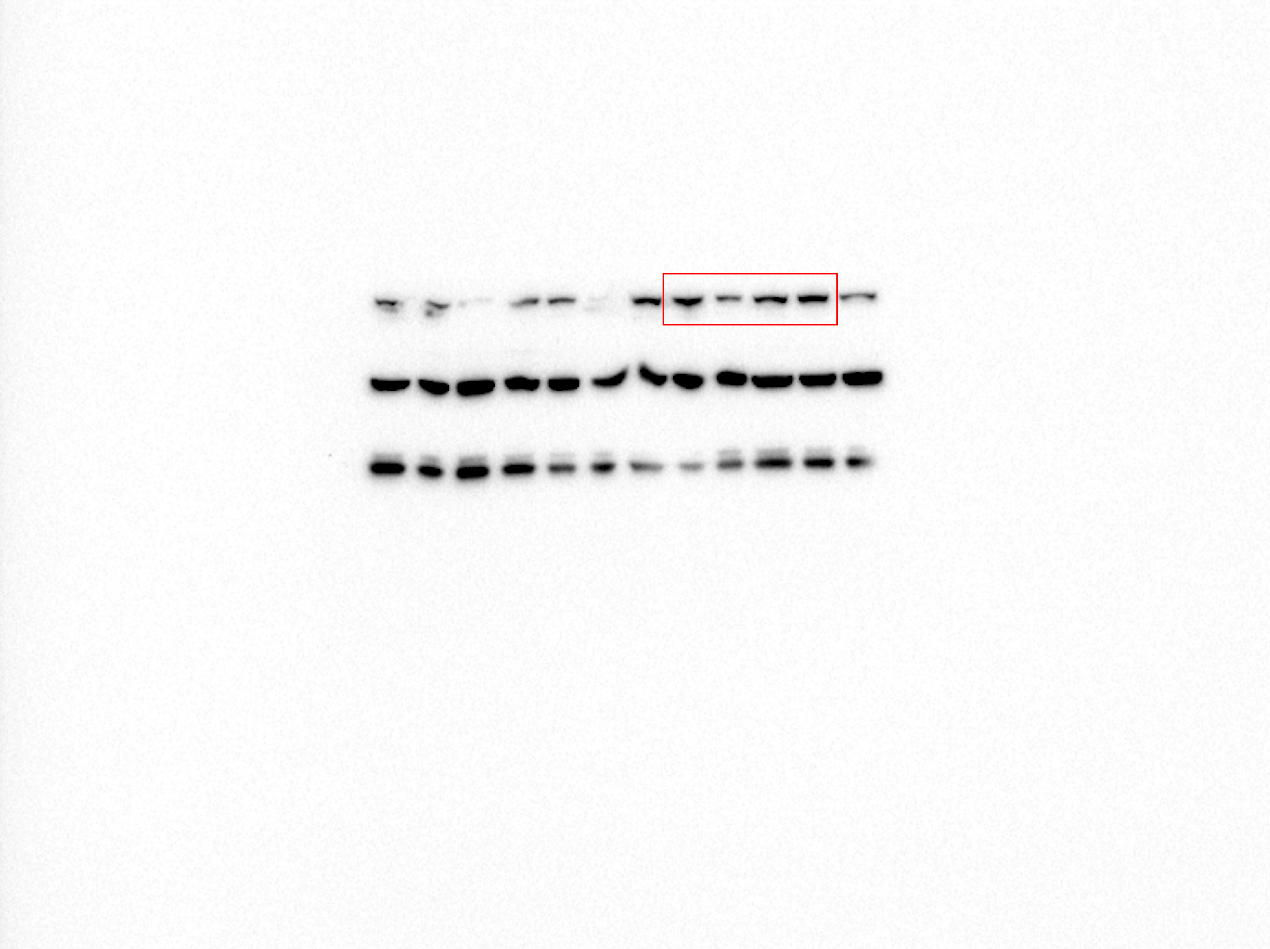


Fig. 4 (A) Protein expression of β-catenin, cyclin D1 and LEF-1 under VDR overexpression and interference conditions in SW480 cells. Western blotting analysis revealed the protein expression of β-catenin under VDR overexpression and interference conditions normalized to GAPDH.

Supplementary fig. 2


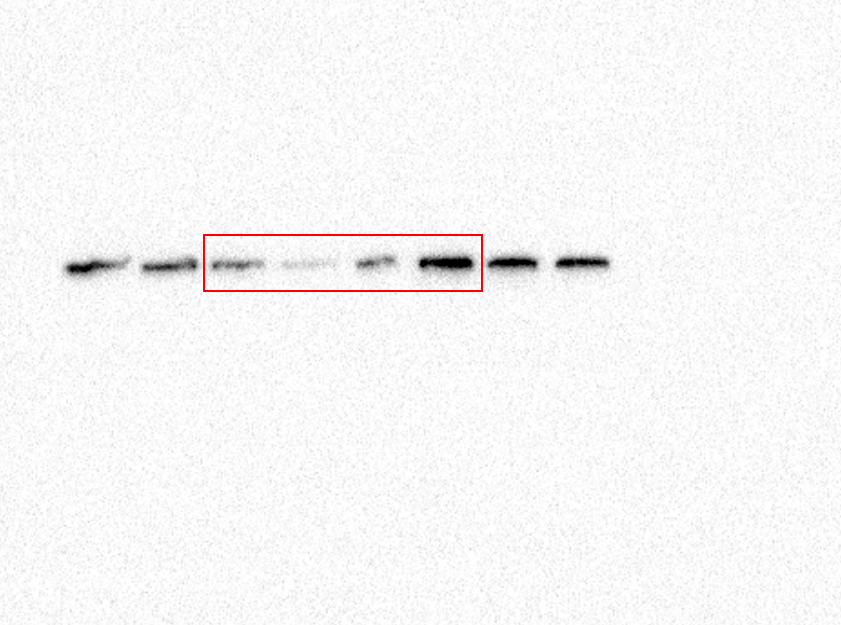


Fig. 4 (A) Protein expression of β-catenin, cyclin D1 and LEF-1 under VDR overexpression and interference conditions in SW480 cells. Western blotting analysis revealed the protein expression of cyclin D1 under VDR overexpression and interference conditions normalized to GAPDH.

Supplementary fig. 3


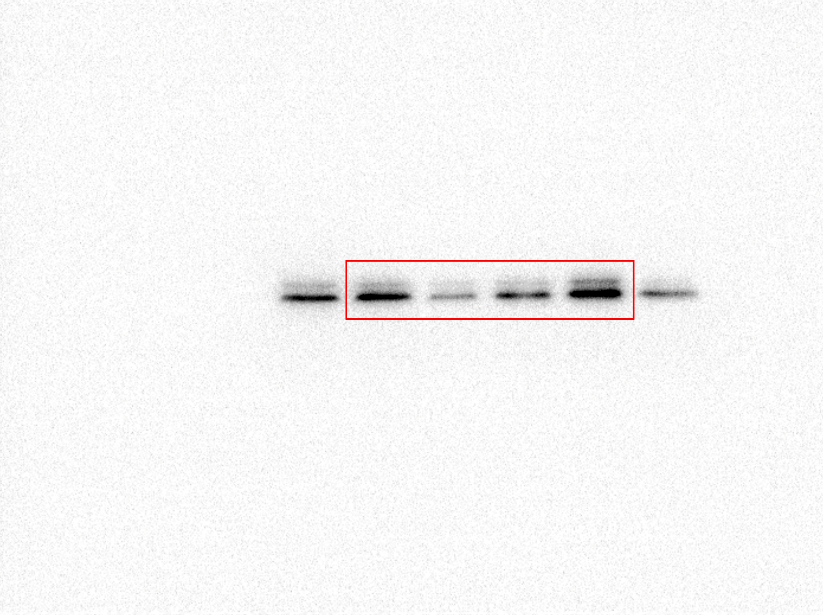


Fig. 4 (A) Protein expression of β-catenin, cyclin D1 and LEF-1 under VDR overexpression and interference conditions in SW480 cells. Western blotting analysis revealed the protein expression of LEF-1 under VDR overexpression and interference conditions normalized to GAPDH.

Supplementary fig. 4


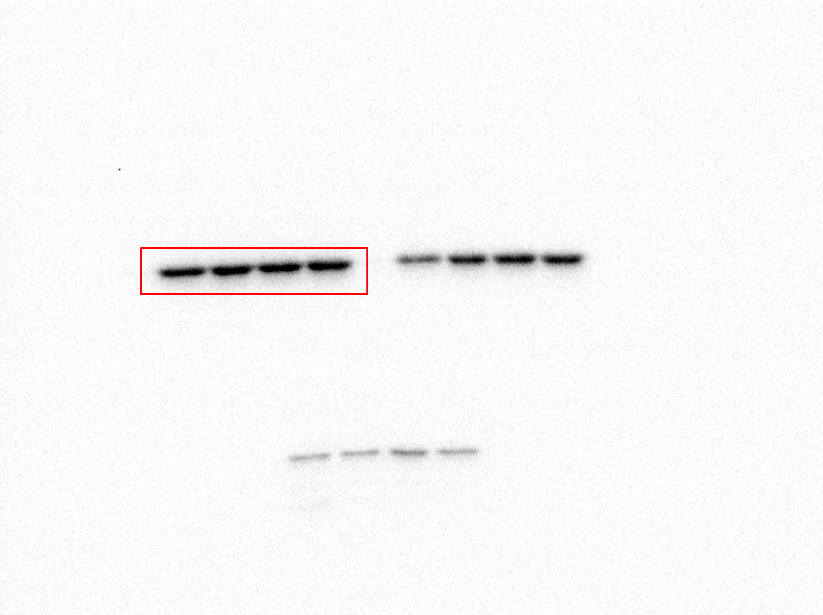


Fig. 4 (A) Protein expression of β-catenin, cyclin D1 and LEF-1 under VDR overexpression and interference conditions in SW480 cells. Western blotting analysis revealed the protein expression of GAPDH under VDR overexpression.

Supplementary fig. 5


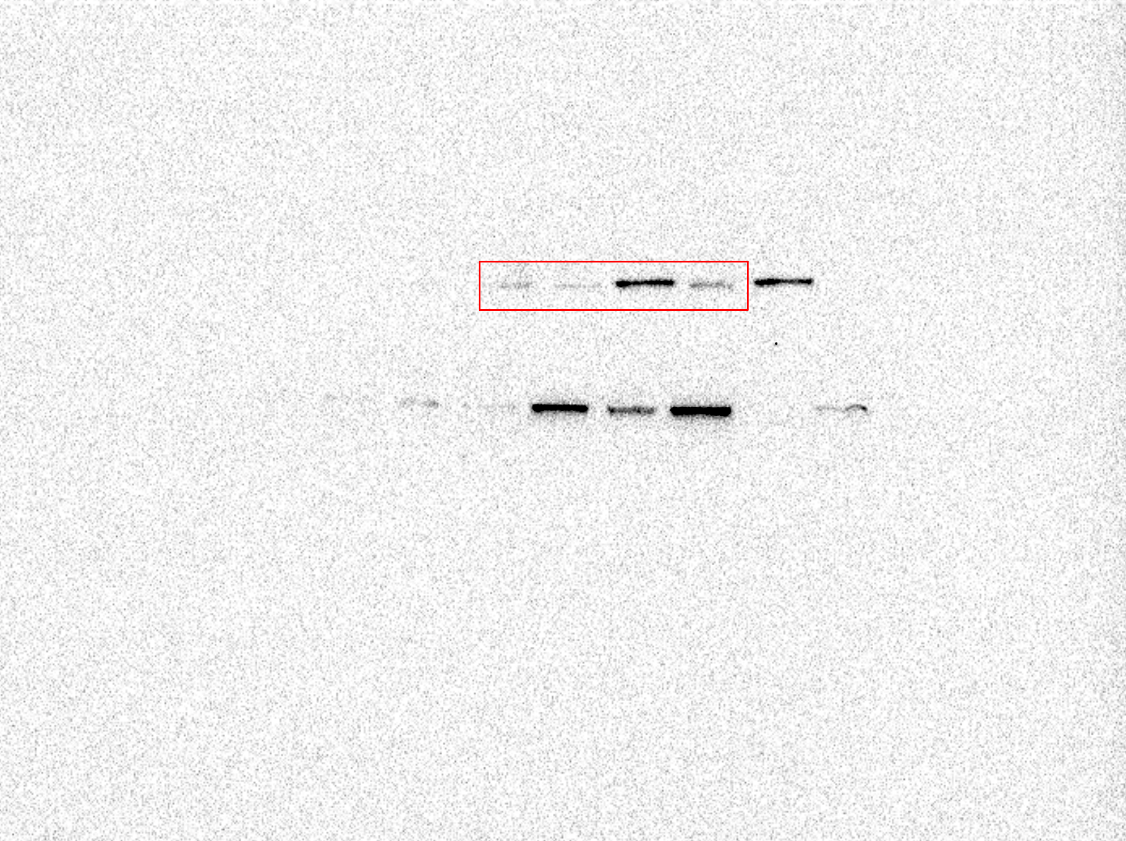


Fig. 6 (A) The Co-IP assay showed that the β-catenin antibody efficiently immunoprecipitated the β-catenin proteins controlled by the input. The initial biomasses were normalized to β-Actin.

Supplementary fig. 6


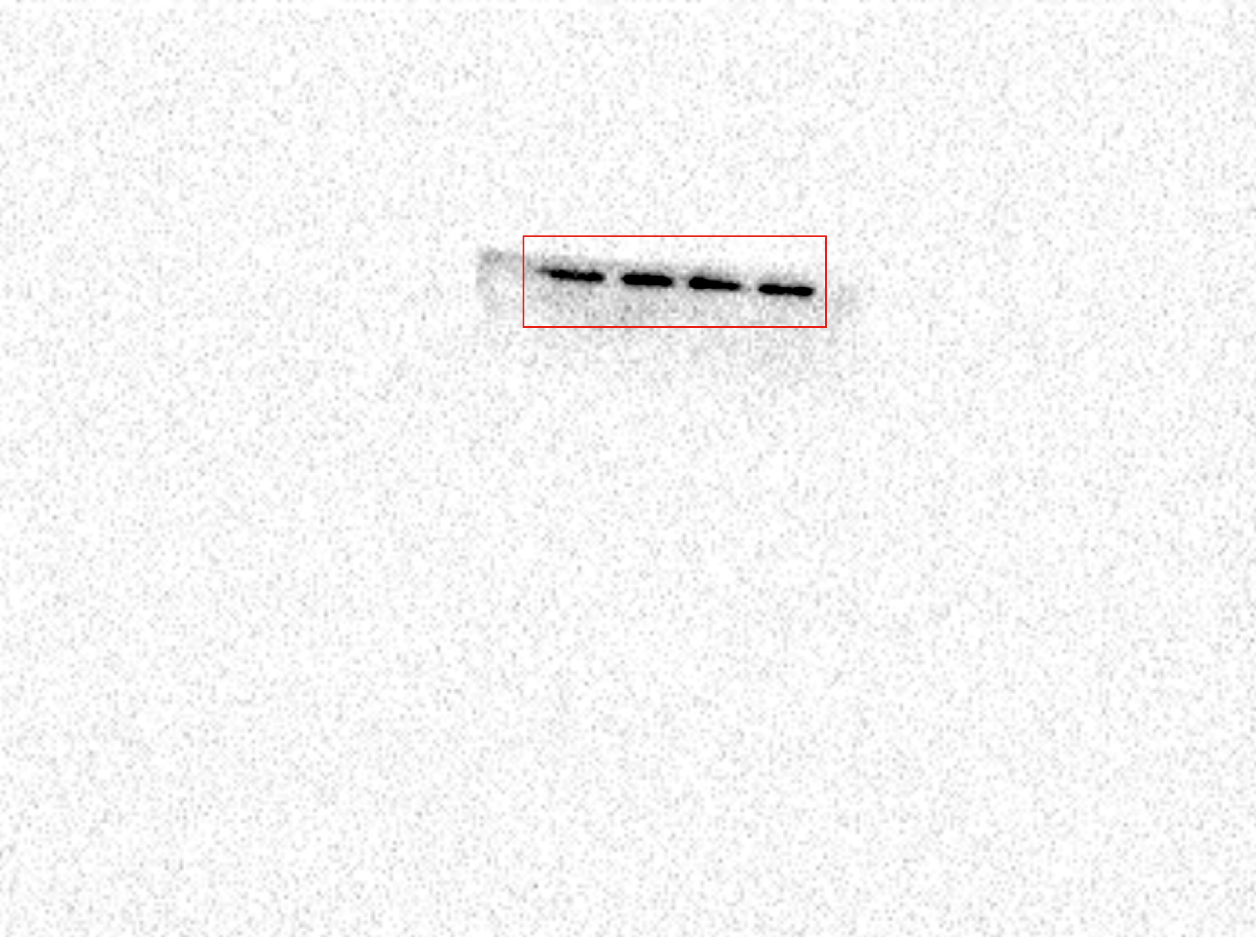


Fig. 6 (A) The Co-IP assay showed that the Myc antibody efficiently immunoprecipitated the β-catenin proteins controlled by the input. The initial biomasses were normalized to β-Actin.

Supplementary fig. 7


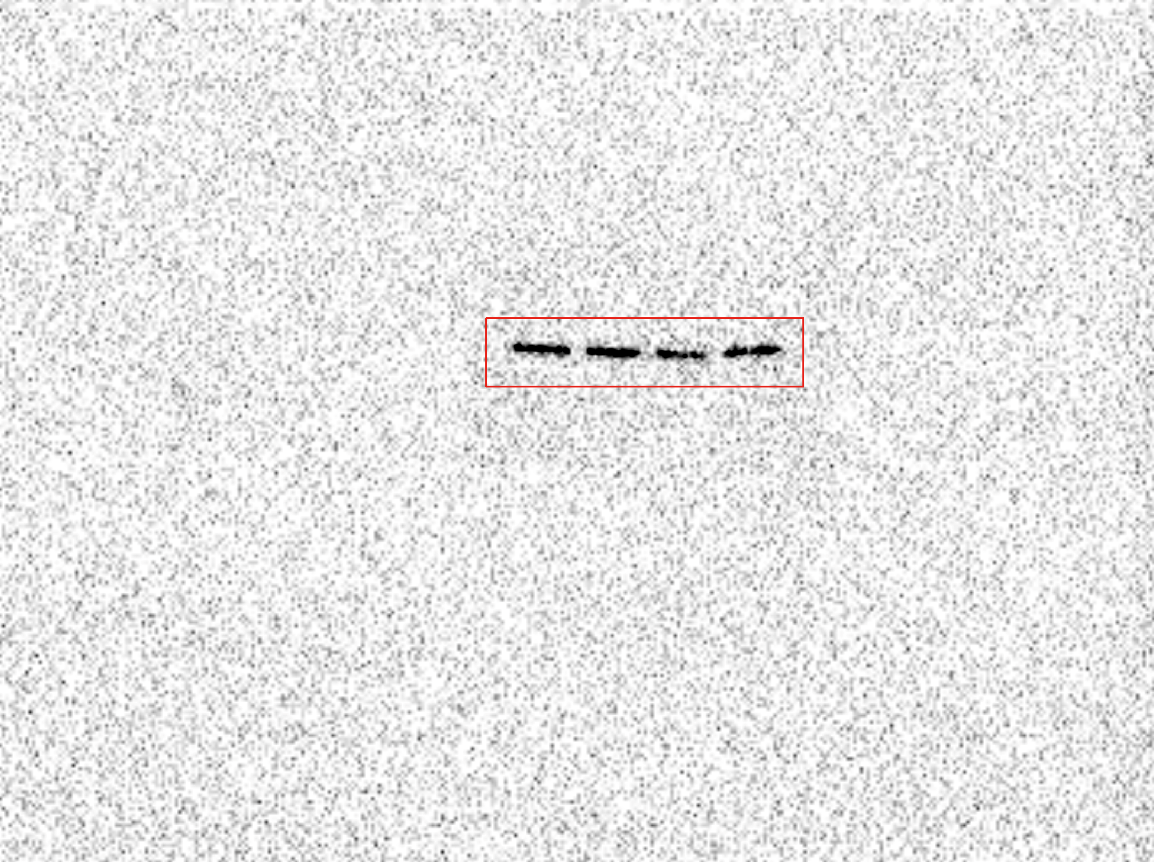


Fig. 6 (A) The Co-IP assay showed that the Myc antibody efficiently immunoprecipitated the Myc proteins. The initial biomasses were normalized to β-Actin.

Supplementary fig. 8


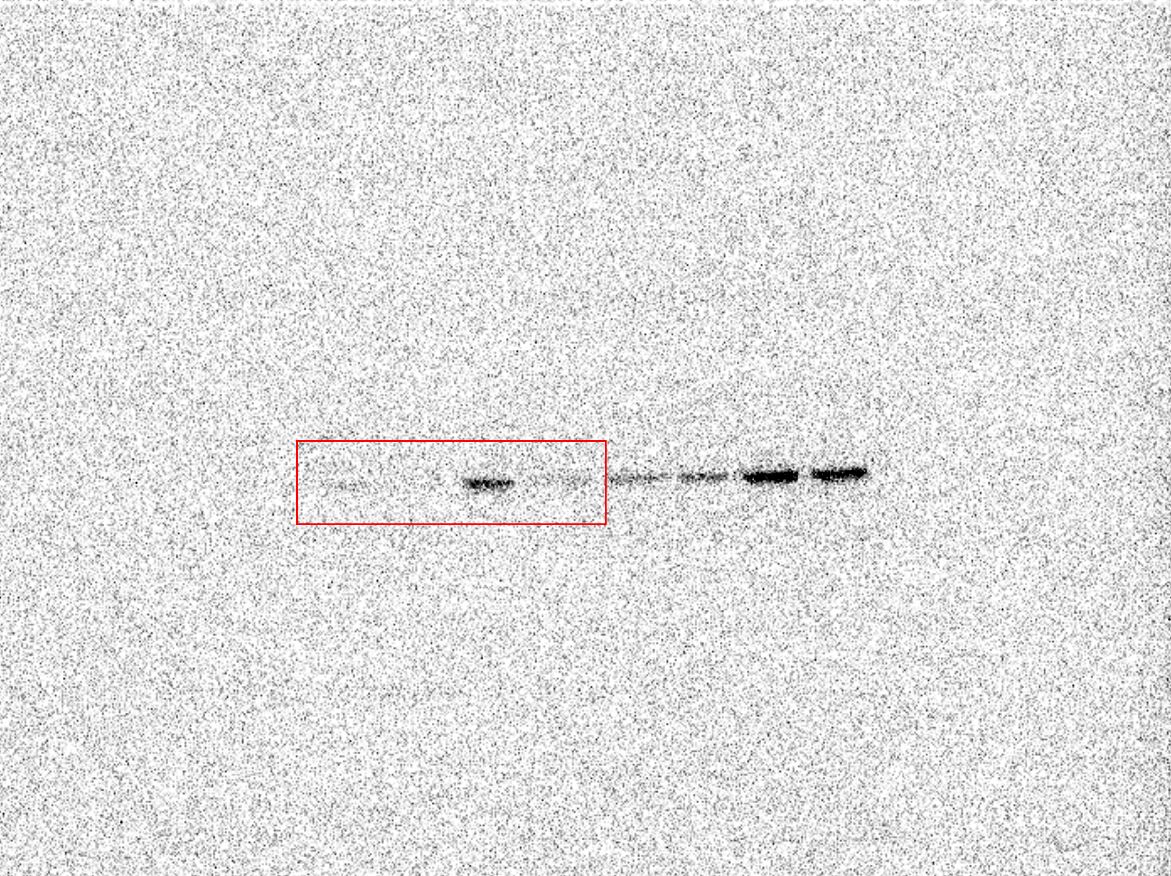


Fig. 6 (A) The Co-IP assay showed that the β-catenin antibody efficiently immunoprecipitated the Myc proteins. The initial biomasses were normalized to β-Actin.

Supplementary fig. 9


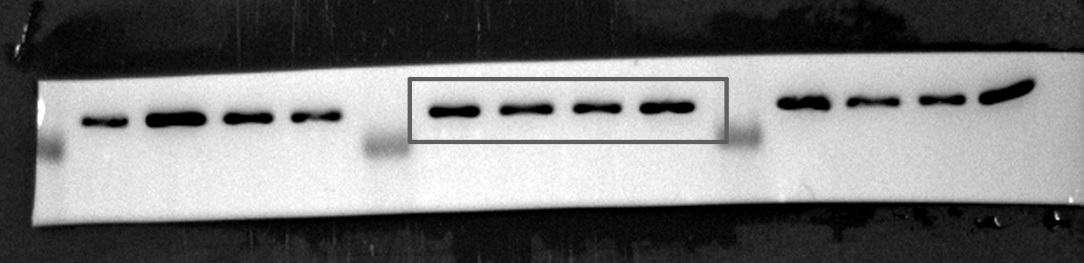


Fig. 6 (A) The Co-IP assay showed the initial biomasses were normalized to β-Actin.

Supplementary fig. 10


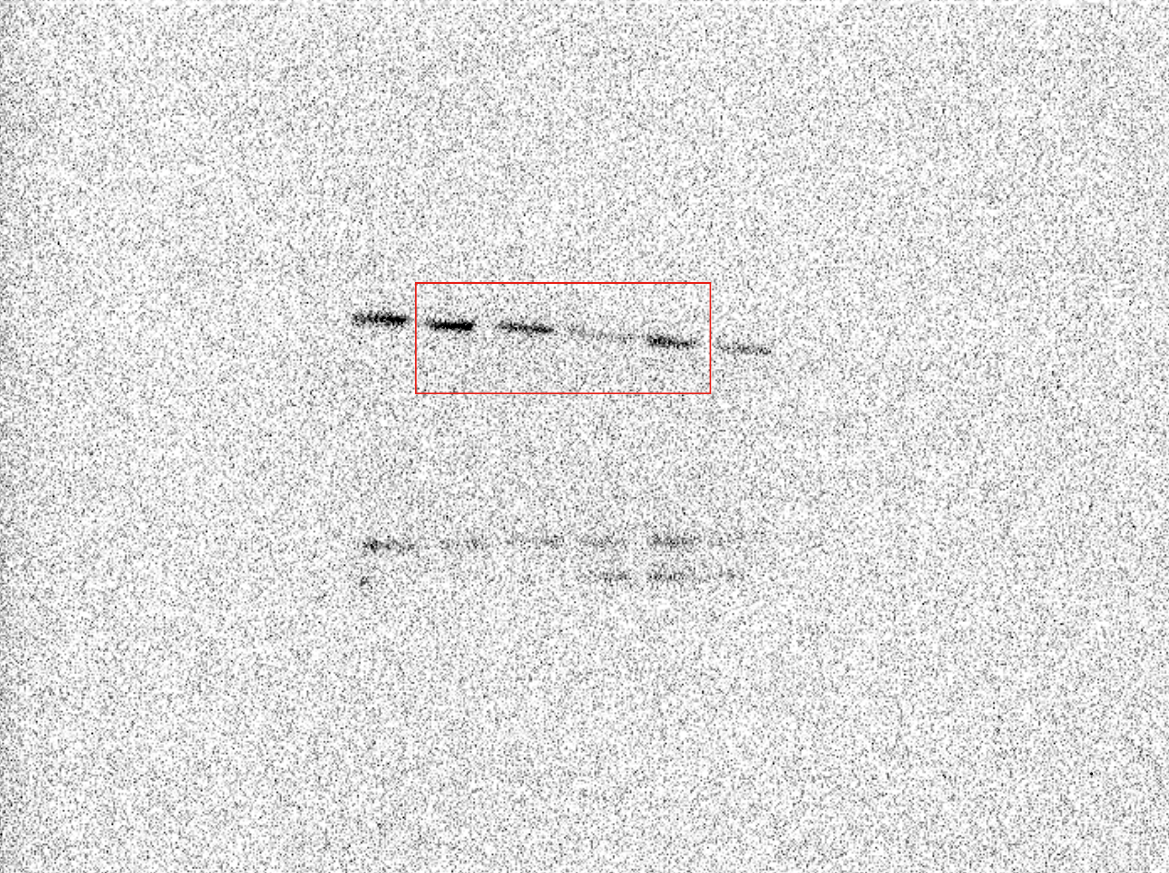


Fig. 6 (B) Western blotting analysis showed the accumulation of β-catenin in the cytoplasm and nucleus with VDR overexpression.

Supplementary fig. 11


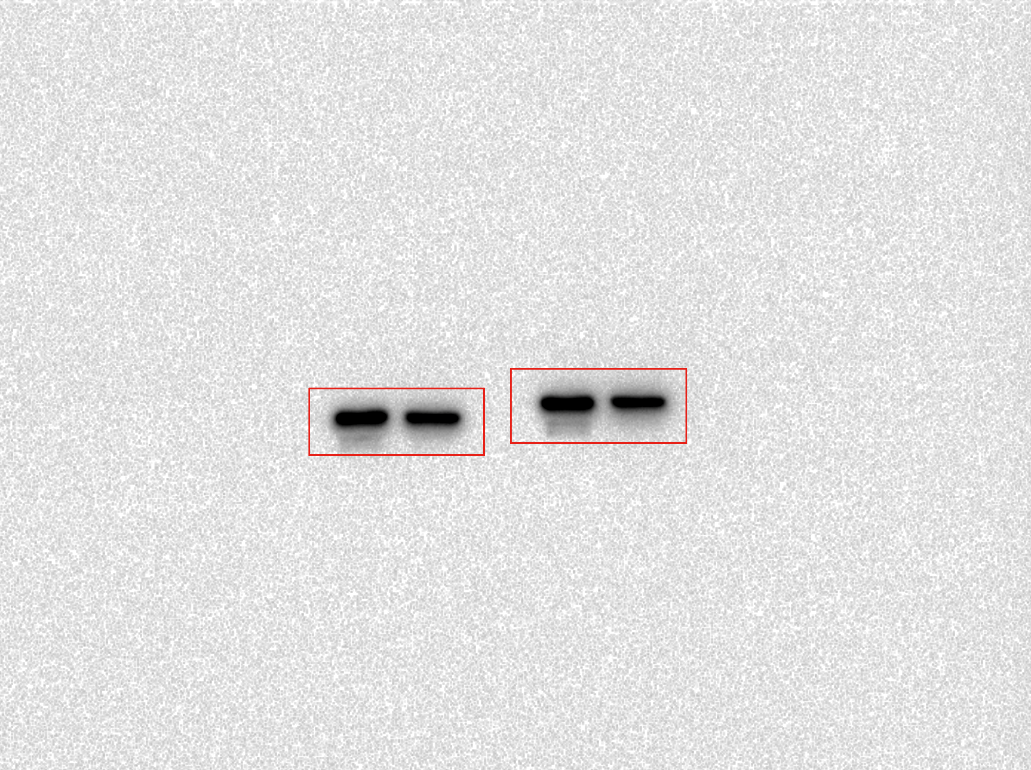


Fig. 6 (B) GAPDH and CREB were used as protein controls in the cytoplasm and nucleus with VDR overexpression.

Supplementary fig. 12


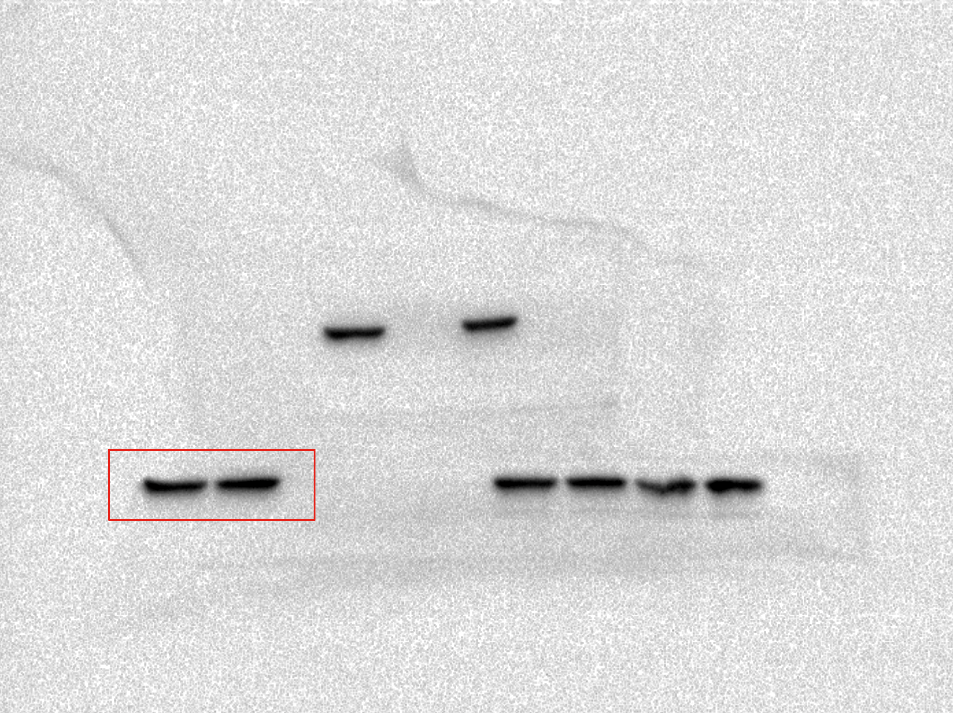


Fig. 6 (C) β-Actin was used as the protein control in the GST pull-down assay.

Supplementary fig. 13


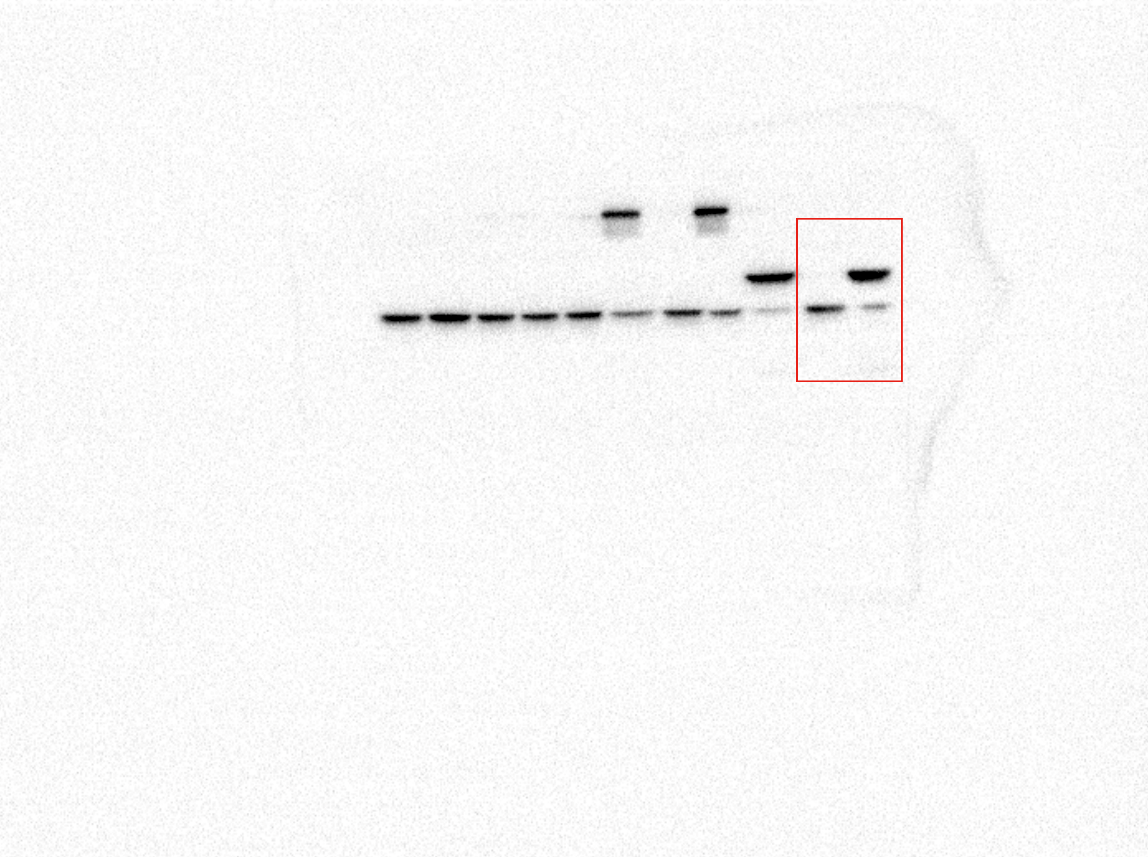


Fig. 6 (C) GST proteins were detected in the GST pull-down assay.

Supplementary fig. 14


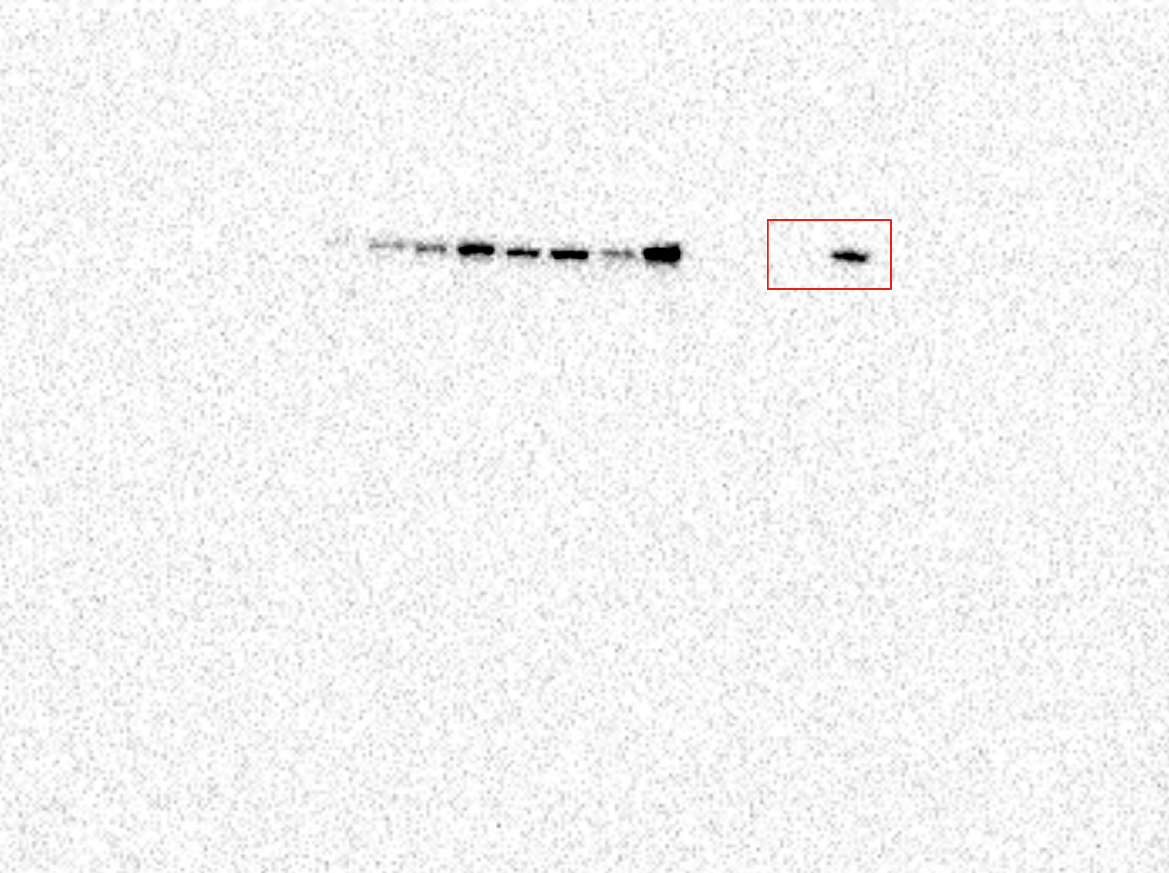


Fig. 6 (C) Myc proteins were detected in the GST pull-down assay.

Supplementary fig. 15


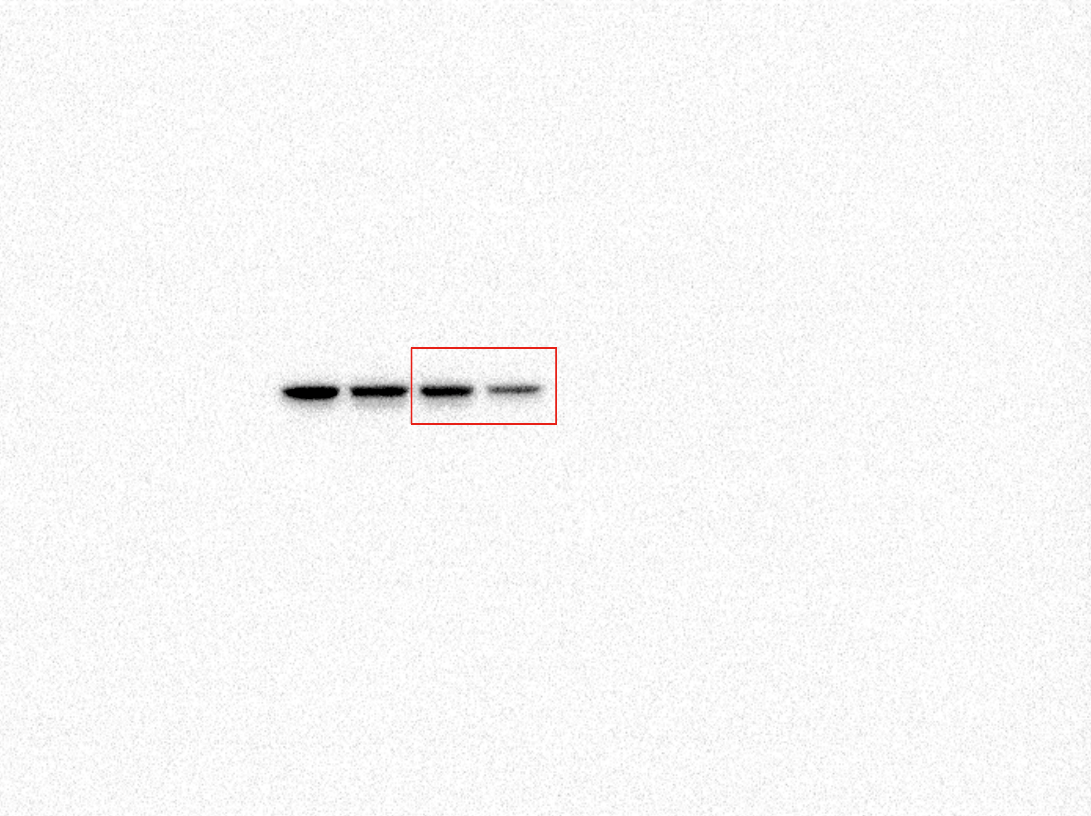


Fig. 8 (D) Western blotting analysis showed the protein levels of β-catenin in the mice of tumours.

Supplementary fig. 16


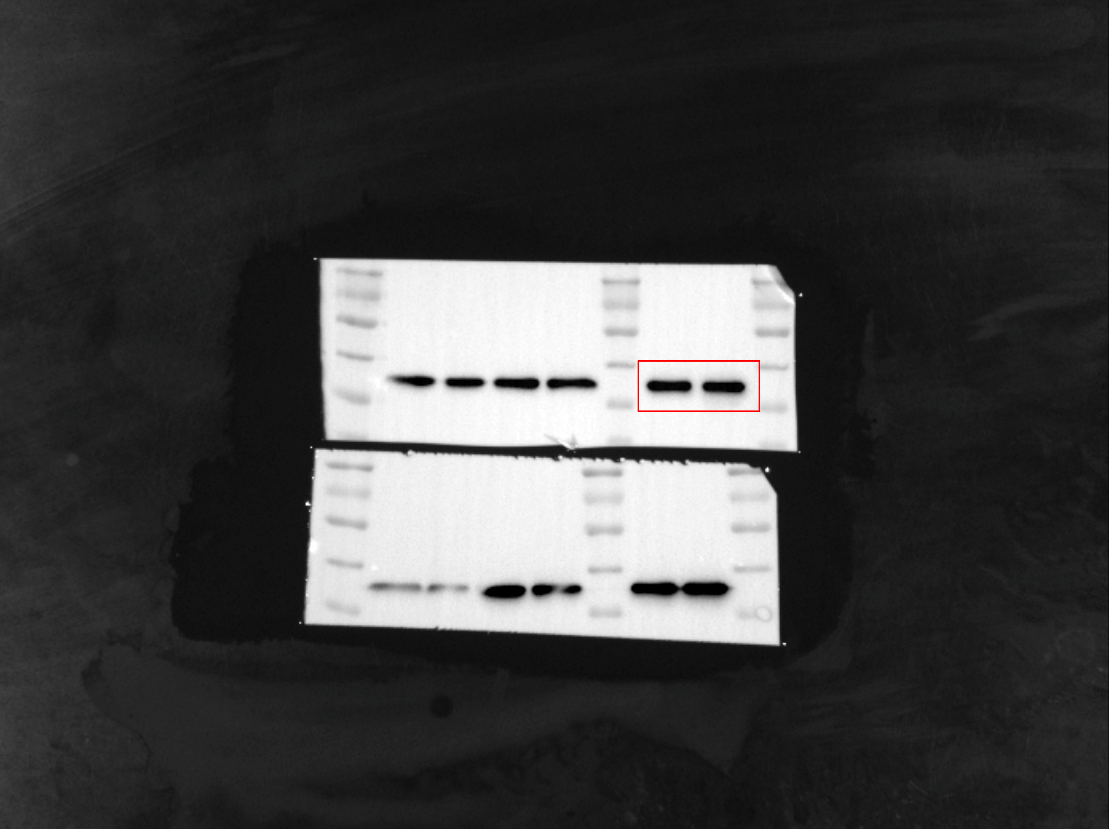


Fig. 8 (D) Western blotting analysis showed the protein levels of GAPDH in the mice of tumours.

Supplementary fig. 17


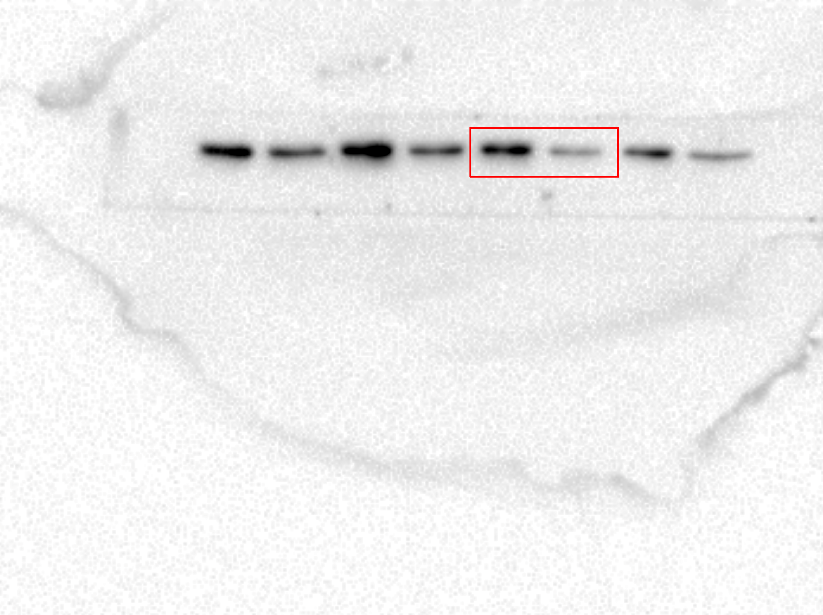


Fig. 8 (D) Western blotting analysis showed the protein levels of CyclinD1 in the mice of tumours.

Supplementary fig. 18


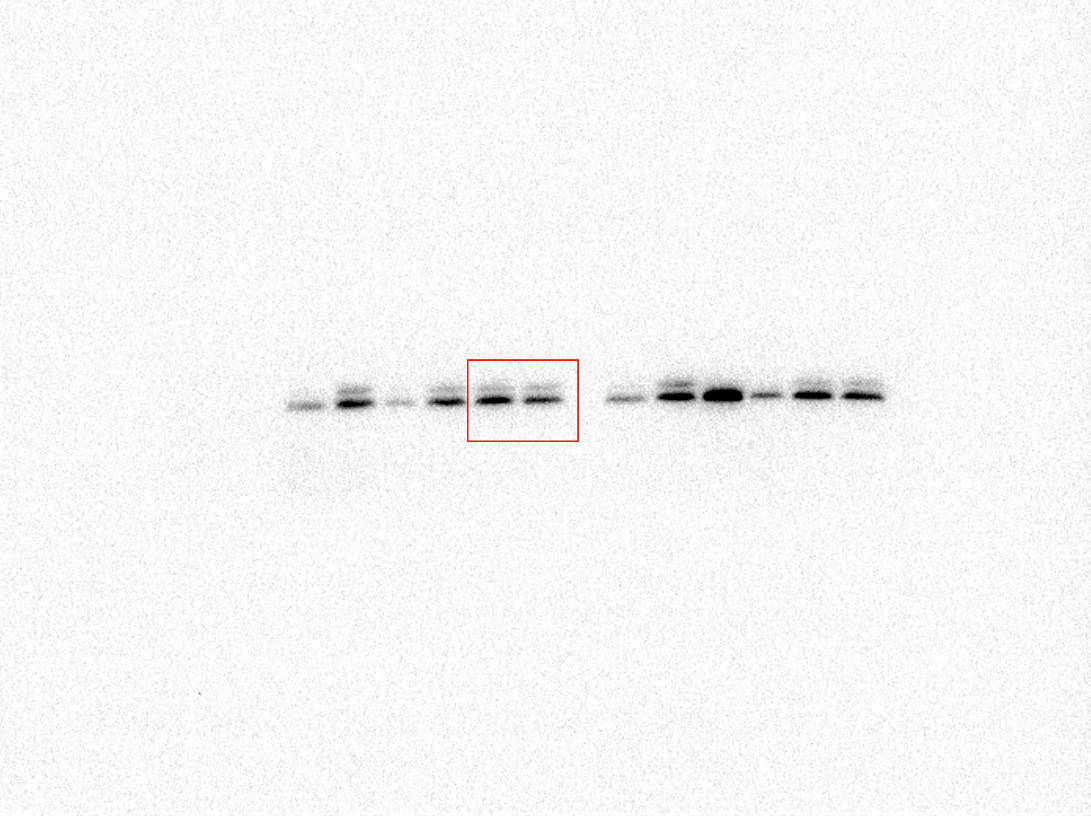


Fig. 8 (D) Western blotting analysis showed the protein levels of LEF-1 in the mice of tumours.

Supplementary fig. 19


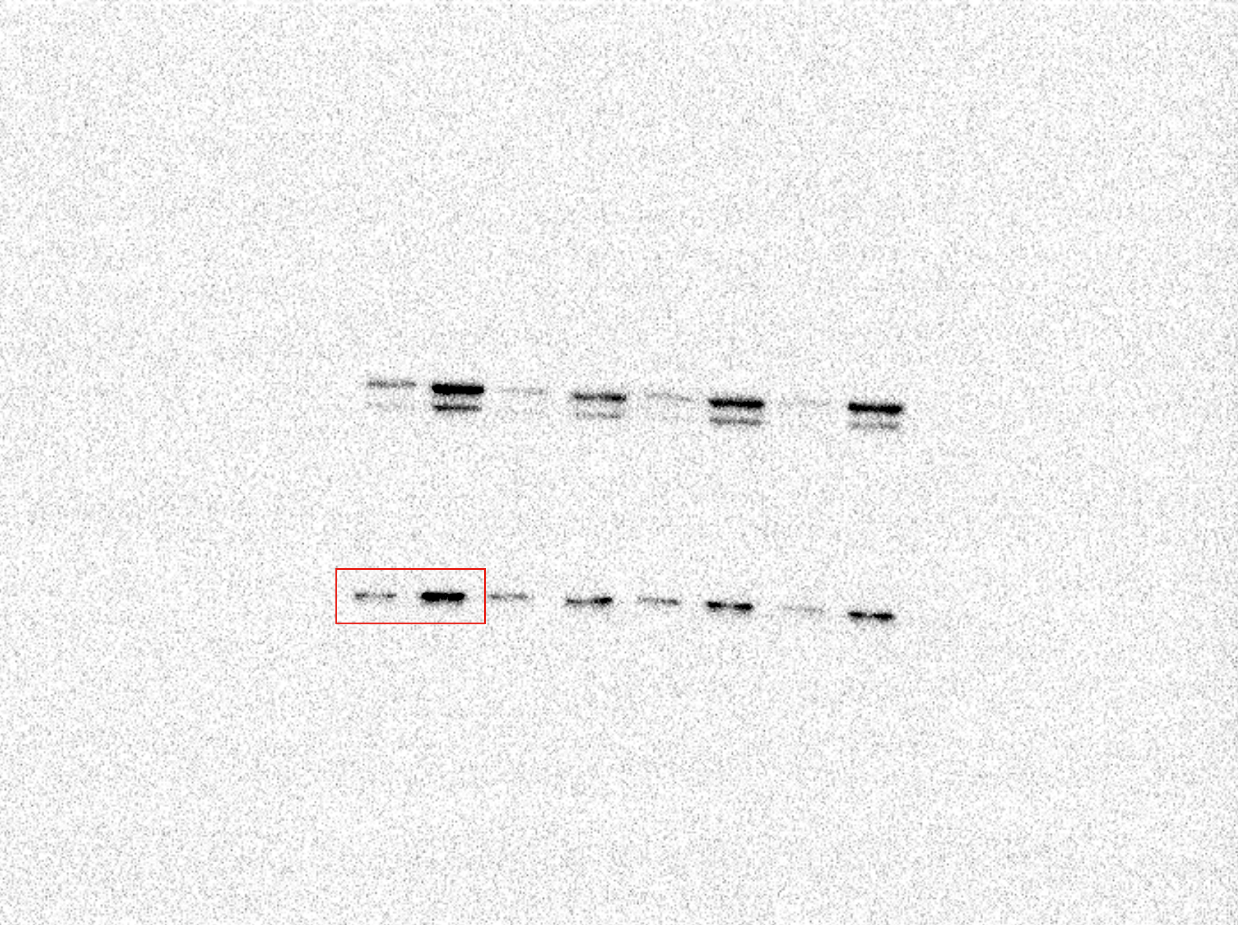


Fig. 8 (D) Western blotting analysis showed the protein levels of VDR in the mice of tumours.

Supplementary fig. 20


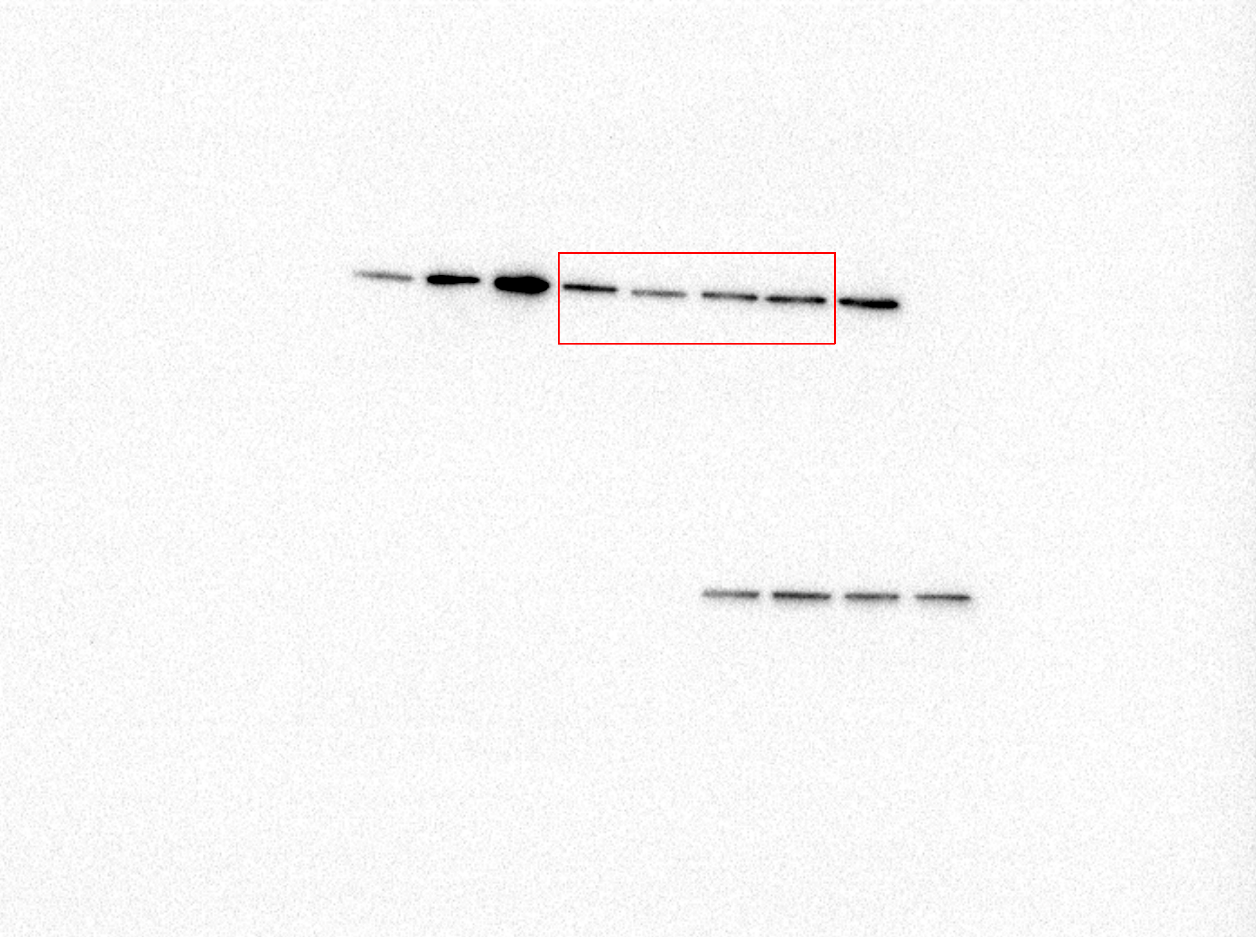


Figure S1 (B) Western blotting analysis of VDR expression in the three different shRNAs.

Supplementary fig. 21


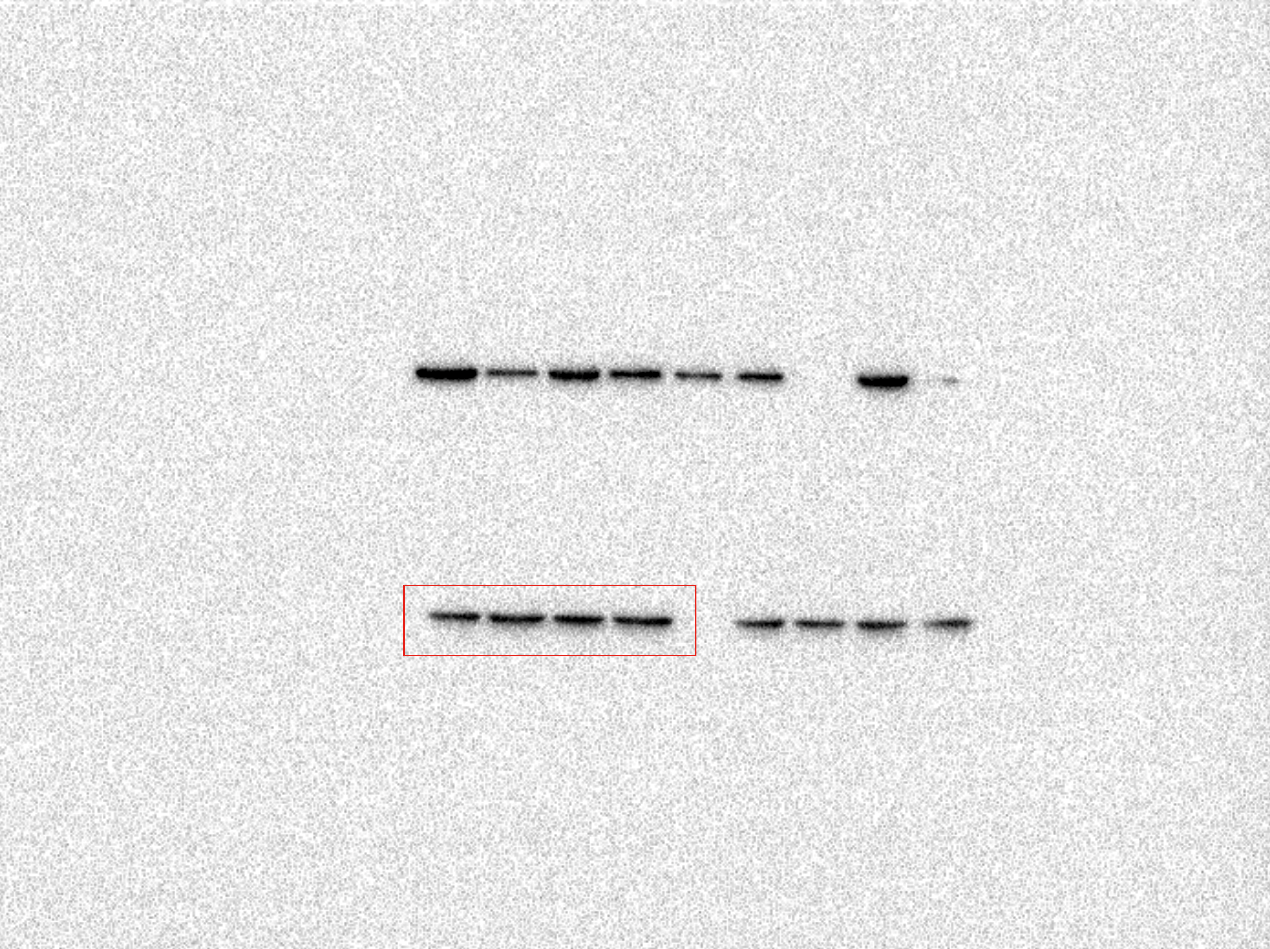


Figure S1 (B) Western blotting analysis of GAPDH expression in the three different shRNAs.

Supplementary fig. 22


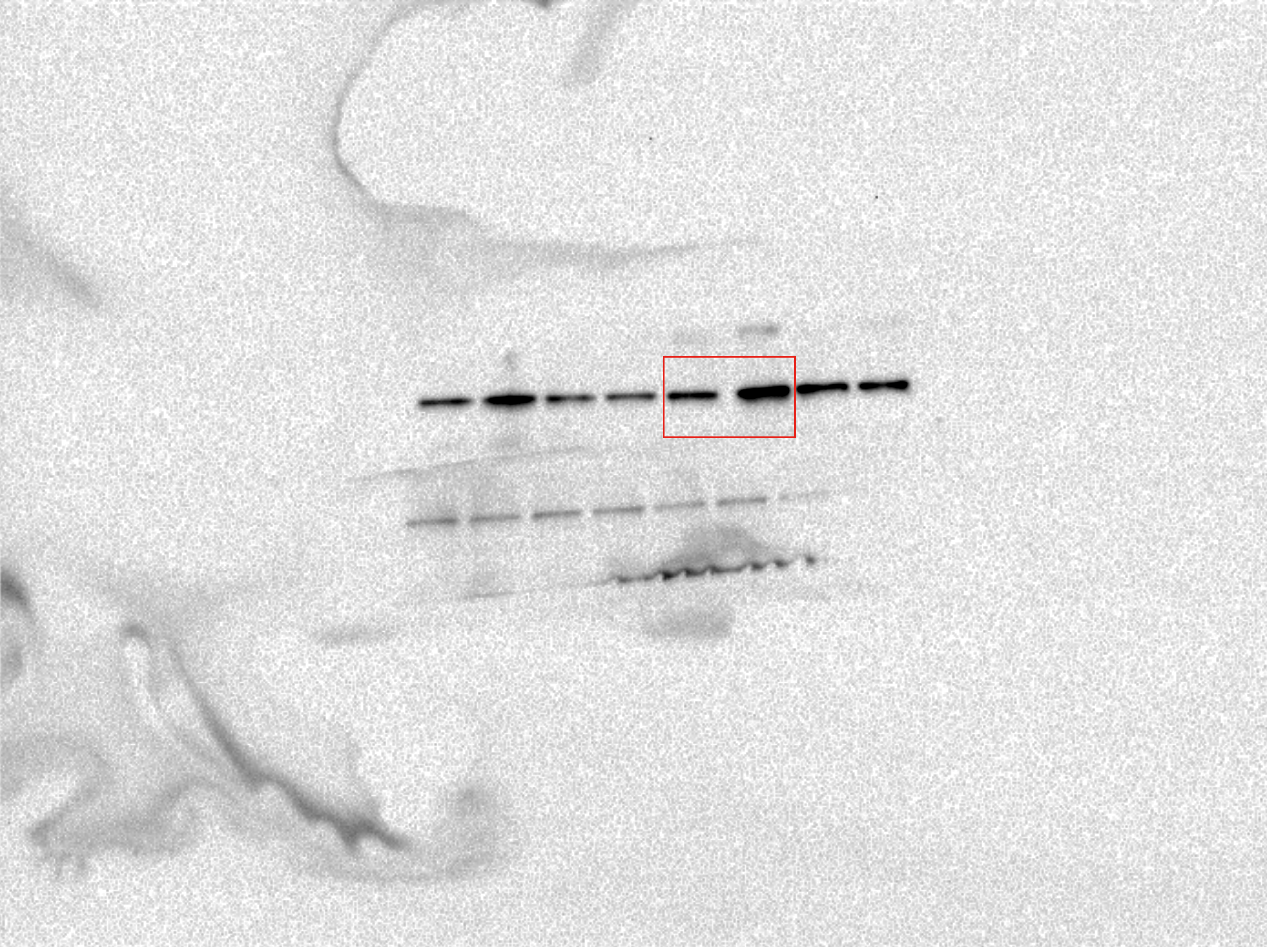


Figure S2 (F) Western blotting analysis of VDR expression in OE-VDR.

Supplementary fig. 23


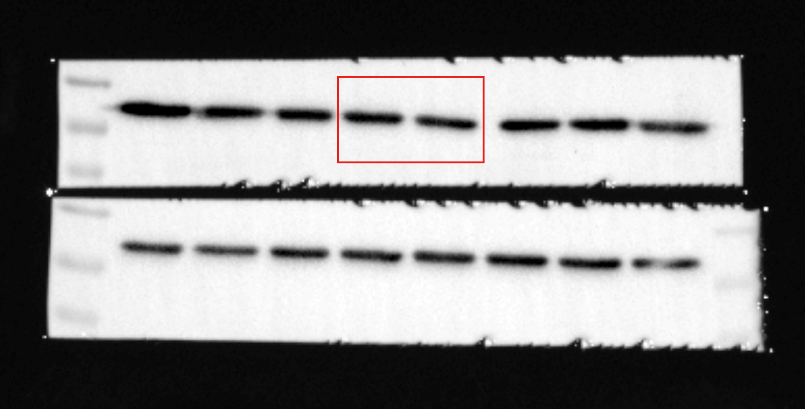


Figure S2 (F) Western blotting analysis of GAPDH expression in OE-VDR.

Supplementary fig. 24


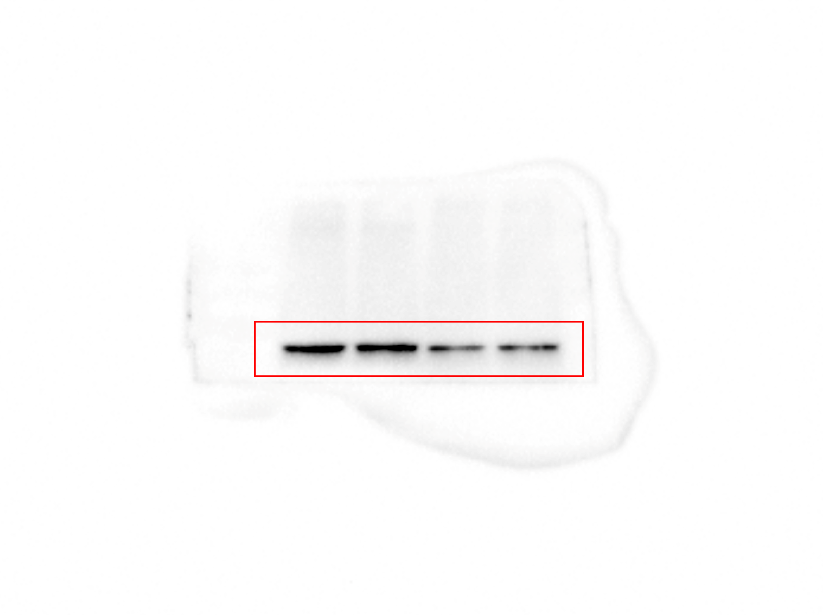


Figure S3. Western blotting analysis of β-catenin expression in the three different shRNAs.

Supplementary fig. 25





Figure S3. Western blotting analysis of GAPDH expression in the three different shRNAs.
